# Nuclear respiratory factor‐1 (NRF1) induction as a powerful strategy to deter mitochondrial dysfunction and senescence in mesenchymal stem cells

**DOI:** 10.1111/acel.14446

**Published:** 2024-12-25

**Authors:** Hyunho Lee, Matteo Massaro, Nourhan Abdelfattah, Gherardo Baudo, Haoran Liu, Kyuson Yun, Elvin Blanco

**Affiliations:** ^1^ Department of Nanomedicine Houston Methodist Research Institute Houston Texas USA; ^2^ Department of Neurology Houston Methodist Research Institute Houston Texas USA; ^3^ Department of Neurology Weill Cornell Medical College New York New York USA; ^4^ Department of Medicine Weill Cornell Medical College New York New York USA; ^5^ Department of Cardiology Houston Methodist DeBakey Heart and Vascular Center, Houston Methodist Hospital Houston Texas USA

**Keywords:** mesenchymal stem cells, mitochondrial biogenesis, mitochondrial dysfunction, nuclear respiratory factor‐1 (NRF1), oxidative stress, senescence

## Abstract

Mesenchymal stem cells (MSCs) are promising candidates for regenerative therapies due to their self‐renewal and differentiation capabilities. Pathological microenvironments expose MSCs to senescence‐inducing factors such as reactive oxygen species (ROS), resulting in MSC functional decline and loss of stemness. Oxidative stress leads to mitochondrial dysfunction, a hallmark of senescence, and is prevalent in aging tissues characterized by elevated ROS levels. We hypothesized that overexpression of nuclear respiratory factor‐1 (NRF1), a driver of mitochondrial biogenesis, could metabolically potentiate MSCs and prevent MSC senescence. Single‐cell RNA sequencing (scRNA‐Seq) revealed that MSCs transfected with NRF1 messenger RNA (mRNA) exhibited upregulated expression of genes associated with oxidative phosphorylation (OXPHOS), decreased glycolytic markers, and suppression of senescence‐related pathways. To test whether NRF1 induction could mitigate stress‐induced premature senescence, we exposed MSCs to hydrogen peroxide (H_2_O_2_) and validated our findings in a replicative senescence model. NRF1 mRNA transfection significantly increased mitochondrial mass and improved aberrant mitochondrial processes associated with senescence, including reduced mitochondrial and intracellular total ROS production. Mitochondrial health and dynamics were preserved, and respiratory function was restored, as evidenced by enhanced OXPHOS, reduced glycolysis, and increased ATP production. Notably, NRF1 overexpression led to decreased senescence‐associated β‐galactosidase (SA‐β‐gal) activity and reduced expression of senescence markers p53, p21, and p16. Our findings demonstrate that NRF1 induction attenuates MSC senescence by enhancing mitochondrial function, suggesting potential translational applications for MSC‐based therapies and senescence‐targeted interventions.

## Graphical abstract

Mitochondrial dysfunction is associated with senescence, the latter proving detrimental to mesenchymal stem cell (MSC)‐based strategies. We induced the expression of nuclear respiratory factor‐1 (NRF1), a driver of mitochondrial biogenesis, to metabolically potentiate MSCs and stave off senescence. This study demonstrates that reinforcement of proper mitochondrial function protects MSCs from senescence.

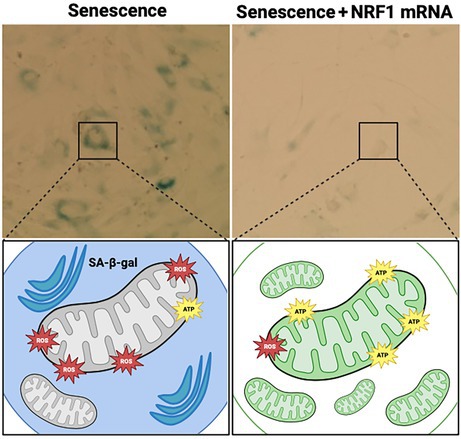

**Abbreviation List**
AcGFP1: aequorea coerulescens green fluorescent protein 1
ATP: adenosine triphosphate
CDKN1A: cyclin‐dependent kinase inhibitor 1A
CDKN2A: cyclin‐dependent kinase inhibitor 2A
COXIV: subunit IV of cytochrome c oxidase
DAPI: 4′,6‐diamidino‐2‐phenylindole
Drp1: dynamin‐related protein 1
ECAR: extracellular acidification rate
EV: extracellular vesicle
FBS: fetal bovine serum
Fis1: fission 1
GSEA: gene set enrichment analysis
H_2_O_2_
: hydrogen peroxide
HIF1A: hypoxia‐inducible factor 1‐alpha
HK2: hexokinase 2
HRP: horseradish peroxidase
JAG1: notch ligand Jagged1
JC‐1: 5,5′,6,6′‐tetrachloro‐1,1′,3,3′‐tetraethylbenzimidazolylcarbocyanine iodide
LAMP1: lysosome‐associated membrane protein 1
LAMP2: lysosome‐associated membrane protein 2
LDHA: lactate dehydrogenase A
MAPK: mitogen‐activated protein kinase
MCT4: monocarboxylate transporter 4
MedFI: median fluorescence intensity
MFC: mitochondrial fragmentation count
MFI: median fluorescence intensity
Mfn2: mitofusin 2
MOI: multiplicity of infection
mRNA: messenger RNA
MSC: mesenchymal stem cell
mtDNA: mitochondrial DNA
NAD: nicotinamide adenine dinucleotide
NADH: nicotinamide adenine dinucleotide reduced
NCPD: number of cell population doubling
NRF1: nuclear respiratory factor‐1
NT: non‐transected
OCR: oxygen consumption rate
OPA1: optic atrophy 1
OXPHOS: oxidative phosphorylation
p5: passage 5
p10: passage 10
PBS: phosphate buffer saline
PCA: principal component analysis
PCR: polymerase chain reaction
PFA: paraformaldehyde
PFKFB3: 6‐phosphofructo‐2‐kinase/fructose‐2,6‐bisphosphatase 3
PGC‐1α: proliferator–activated receptor gamma coactivator‐1α
ROS: reactive oxygen species
RT: room temperature
RT‐qPCR: reverse transcription‐quantitative PCR
SASP: senescence‐associated secretory phenotype
SA‐β‐gal: senescence‐associated β‐galactosidase
SCP: statistical computing and graphics
SCR: scrambled mRNA
scRNA‐Seq: single‐cell RNA sequencing
SDS‐PAGE: sodium dodecyl sulfate polyacrylamide gel electrophoresis
SEM: standard error of the mean
SGJ: 3‐butyl‐1‐chloro imidazo [1, 5‐a] pyridine‐7‐carboxylic acid
SIRT1: sirtuin 1
TAZ: transcriptional coactivator with PDZ‐binding motif
TBST: tris‐buffered saline 0.1% Tween 20
TFAM: mitochondrial transcription factor A
TP53: tumor protein p53
TRITC: tetramethylrhodamine isothiocyanate
X‐gal: 5‐bromo‐4‐chloro‐3‐indolyl beta‐d‐galactopyranoside
ΔΨm: mitochondrial membrane potential

## 1 INTRODUCTION

Mesenchymal stem cell (MSC)‐based therapies represent a cornerstone of regenerative medicine. Advantages afforded by MSCs include their availability in a variety of tissues (e.g., adipose and bone marrow), their propensity for self‐regeneration and proliferation, and their ability to differentiate into varied end‐stage cell lineages, including chondrocytes and osteoblasts (Han et al., 2019). Specifically, bone marrow‐derived MSCs exhibit a heightened propensity for osteogenesis and chondrogenesis, making them invaluable in tissue engineering and repair applications, including the reconstruction of nervous and musculoskeletal systems, as well as cardiac and liver tissues. Moreover, MSCs modulate both innate and adaptive immunity through interactions with immune cells like T cells, B cells, monocytes, macrophages, neutrophils, natural killer cells, and dendritic cells (Song et al., 2020). This immunomodulation of the surrounding microenvironment is exerted through cell‐to‐cell contacts and the MSC secretome, the latter consisting of cytokines, chemokines, and growth factors capable of promoting tissue regeneration and impacting inflammatory disease progression. The low immunogenicity of MSCs further facilitates their use in autologous or allogeneic transplantation strategies, contributing to their widespread investigation in clinical trials for conditions such as cardiovascular disease, graft‐versus‐host disease, and multiple sclerosis (Galderisi et al., 2022).

Despite their transformative potential, MSC therapies face significant translational challenges due to senescence (McHugh & Gil, 2018). A preponderant presence and excessive production of reactive oxygen species (ROS) is associated with cancer and the progression of cardiovascular, neurodegenerative, and respiratory diseases (Liu et al., 2018). Oxidative stress is a key driver of senescence, as ROS triggers the DNA damage response network in MSCs, activating the p21^CIP1/WAF1^ and p16^INK4A^ cell cycle arrest pathways (Weng et al., 2022). Notably, oxidative stress results in mitochondrial dysfunction. Processes vital to mitochondrial quality control and homeostasis are impaired in senescent MSCs, and accumulation of damaged mitochondria combined with reduced antioxidant capacity leads to higher ROS levels, resulting in a destructive, self‐perpetuating cycle. Metabolic disturbances abound, including dysregulated cell respiration and respiratory chain defects that give rise to abnormal NAD+/NADH ratios and decreased ATP production (Miwa et al., 2022). Senescence also represents a hallmark of aging, the latter associated with higher ROS generation and an impairment in mitochondrial function (Maldonado et al., 2023). Importantly, the quantities of MSCs required for clinical applications necessitates in vitro expansion involving numerous population doublings, which in turn erodes MSC telomeres and reduces their proliferative capacity and differentiation potential (Liu, Ding, et al., 2020). MSCs isolated from aged donors possess diminished proliferation and differentiation, and display poor migratory and homing capabilities (Baker et al., 2015). Thus, strategies aimed at preserving mitochondrial function may prove beneficial in abrogating MSC senescence.

The objective of this study was to deter MSC senescence by reinforcing mitochondrial function and metabolic priming. We previously showed that exogenous mitochondrial transplantation into cells increased oxidative phosphorylation (OXPHOS) and ATP production, and reduced ROS (Baudo et al., 2023). These findings suggest that increasing mitochondrial mass within cells ensures the continued well‐functioning of mitochondria, even in the face of external stimuli such as oxidative stress. We hypothesized that overexpression of nuclear respiratory factor‐1 (NRF1), an activator of mitochondrial transcription factor A (TFAM) that drives mitochondrial DNA (mtDNA) transcription and replication (Gleyzer et al., 2005; Puigserver & Spiegelman, 2003), would result in preservation of mitochondrial health and metabolic potentiation of MSCs, thereby staving off oxidative stress‐induced senescence. We principally examined this strategy in a stress‐induced premature senescence model, wherein MSCs were exposed to hydrogen peroxide (H_2_O_2_), and validated our findings in a replicative senescence model. Our results demonstrate a significant increase in mitochondrial mass in MSCs following NRF1 mRNA transfection. Upon NRF1 induction in MSCs undergoing senescence, hallmarks of mitochondrial dysfunction (e.g., ROS accumulation) were largely absent and mitochondrial function was preserved. Single‐cell RNA sequencing (scRNA‐Seq) of NRF1‐transfected MSCs demonstrated increased expression of OXPHOS genes, decreased glycolysis markers, and a reduction of senescence‐related gene expression. Importantly, senescence‐associated processes were effectively mired. Findings highlight the potential to counteract MSC senescence through bolstering of mitochondrial function and bioenergetics, effectively addressing a major limitation to MSC‐based therapies for in vivo transplantation.

## 2 RESULTS AND DISCUSSION

### 2.1 NRF1 overexpression increased mitochondrial mass in MSCs undergoing senescence

**FIGURE 1:**
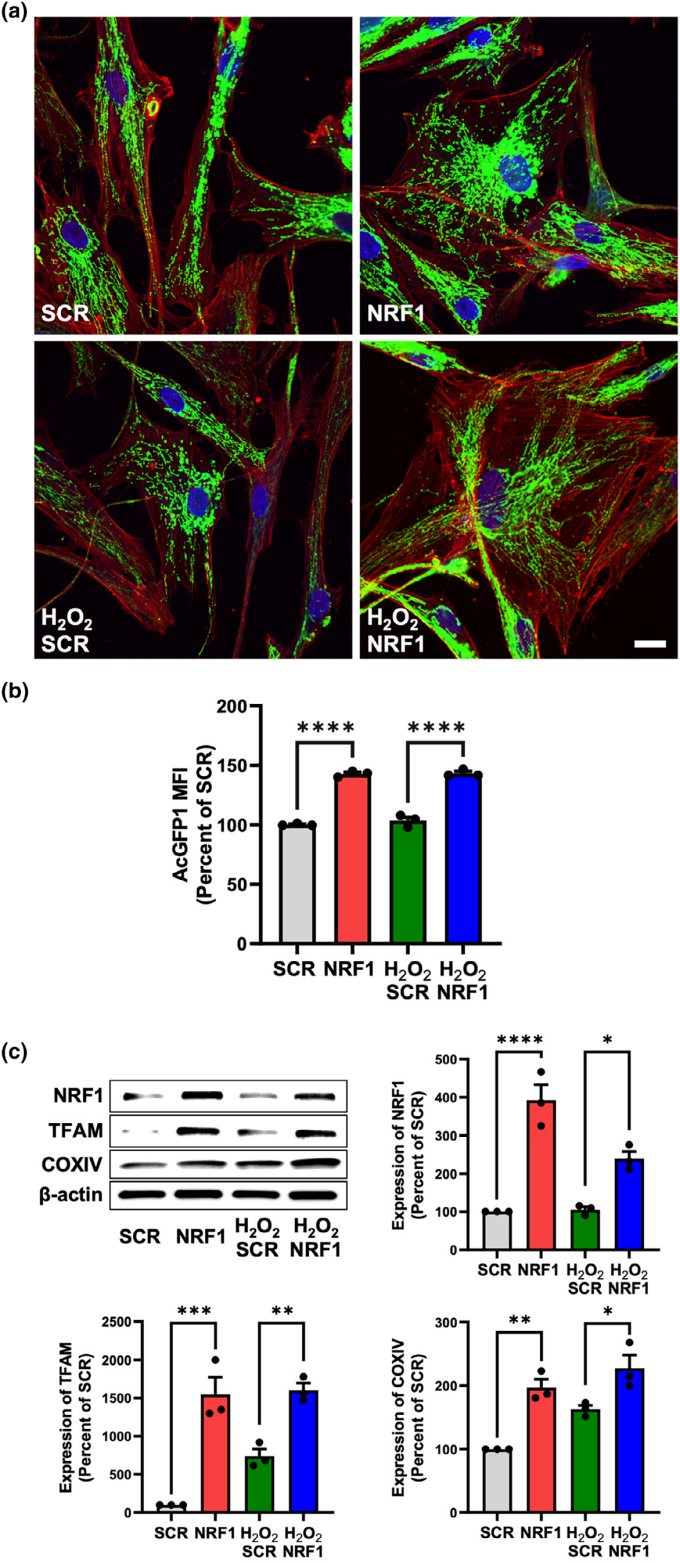
NRF1 overexpression increased mitochondrial mass in MSCs exposed to oxidative stress. Healthy and H_2_O_2_‐exposed (250 μM, 1 h) MSCs were transfected with either scrambled (SCR) or NRF1 mRNA. (a) Representative confocal microscopy images depicting AcGFP1‐expressing mitochondria in MSCs. Green represents AcGFP1‐associated fluorescence, red represents F‐actin staining, and blue represents DAPI nuclear staining. Scale bar = 15 μm. (b) AcGFP1 mean fluorescence intensity (MFI) quantified by flow cytometry analysis. (c) Representative western blot of NRF1, TFAM, and COXIV expression in MSCs. Densitometric analysis was performed to quantify protein expression. Protein markers were normalized to β‐actin expression levels. *p < 0.05; **p < 0.005; ***p < 0.0005; ****p < 0.0001.

Our primary objective was to increase mitochondrial content in MSCs to enhance metabolic capacity and mitochondrial function, thereby increasing MSC resilience against senescence. Mitochondrial biogenesis is primarily driven by peroxisome proliferator‐activated receptor gamma coactivator‐1α (PGC‐1α), which regulates nuclear respiratory factors NRF1 and NRF2 (Puigserver & Spiegelman, 2003). Specifically, PGC‐1α binds to and coactivates the transcriptional activity of NRF1, which in turn activates TFAM, a key regulator of mtDNA replication and transcription (Gleyzer et al., 2005). We aimed to stimulate mitochondrial biogenesis through direct upregulation of NRF1 expression. MSCs transfected with NRF1 mRNA had increased mitochondrial content in cells compared to scrambled (SCR) mRNA controls at a 24‐h timepoint, as evidenced by confocal microscopy analysis and quantitative examination via flow cytometry of mitochondria‐associated fluorescence (AcGFP1) expression (Figure 1a,b). NRF1 expression was significantly increased after NRF1 mRNA transfection compared to SCR mRNA‐treated controls (Figure 1c). TFAM and Subunit IV of cytochrome c oxidase (COXIV), an enzyme whose protein expression serves as an indicator of mitochondrial mass, were also elevated after NRF1 mRNA transfection of MSCs (Figure 1c).

We leveraged our scRNA‐Seq dataset to examine the efficiency of NRF1 mRNA transfection. Feature plots revealed a significant increase in normalized NRF1 expression in NRF1 mRNA‐transfected MSCs compared to non‐transfected (NT) and SCR controls at the single cell level (Figure S1a). Approximately 90% of NRF1 mRNA‐transfected cells exhibited a 28‐ to 35‐fold increase in Log2 NRF1 counts relative to the mean NRF1 expression in the SCR group (Figure S1b). NT and SCR samples had average counts of 0.5 and 0.4, respectively, indicating null NRF1 expression in ~70% of these cells (Figure S1c). In contrast, NRF1 mRNA‐transfected MSCs showed an average of over 3000 raw counts, with only 0.8% of cells displaying null NRF1 expression. NT and SCR samples had negligible NRF1 expression, with <10% of cells showing more than a single count of NRF1 transcripts compared to over 98% of cells in the NRF1 mRNA‐transfected group. Of note, none of the SCR MSCs had counts >7, whereas 96.4% of NRF1 mRNA‐transfected MSCs had counts exceeding 7 (Figure S1c). Remarkably, over 90% of NRF1 mRNA‐transfected MSCs had counts >175, which is 25‐fold higher than the highest counts observed in controls. Taken together, >96% of NRF1 mRNA‐transfected cells overexpressed NRF1, with >90% of cells showing more than 25‐fold increase in NRF1 expression. NRF1 mRNA transfection efficiency was also assessed via RT‐qPCR, with the level of NRF1 mRNA in NRF1 mRNA‐transfected MSCs significantly higher than in SCR MSCs (Figure S2). Despite the decrease in NRF1 mRNA expression observed at 48 and 72 h, mRNA was still significantly higher at these timepoints compared to SCR and NT MSCs. Decline in mRNA levels starting at 48 h may stem from natural cellular RNA decay mechanisms involved in degrading foreign RNA, via ribonucleases or RNases, to maintain cellular homeostasis (Houseley & Tollervey, 2009).

Fluorescence‐based examination showed that mitochondrial content remained significantly increased at 24 and 48 h post‐transfection with NRF1 mRNA, with a decrease observed at 72 h (Figure S3a,b). Protein levels of NRF1, TFAM, and COXIV were stably elevated over the course of 48 h, before also experiencing a decrease at the 72 h timepoint (Figure S3c). Analysis of mtDNA copy number showed a significant increase in mtDNA levels per cell at timepoints of 24 and 48 h compared to SCR controls, with a decrease observed at 72 h (Figure S3d). Lastly, examination of citrate synthase activity supported the presence of increased mitochondrial content due to NRF1 induction, particularly at earlier timepoints of 24 and 48 h (Figure S3e).

H_2_O_2_ gives rise to free radicals and affects cell proliferation and migration, differentiation and survival, gene expression, and cell death (Xin et al., 2022), and was used herein to trigger oxidative stress in MSCs and as a means to induce the stress‐induced premature senescence model. NRF1 mRNA‐induced mitochondrial biogenesis was also observed in MSCs undergoing oxidative stress, with higher mitochondrial content present in NRF1 mRNA‐transfected MSCs compared to SCR controls, as evidenced by both lentiviral‐ (Figure 1a,b) and MitoTracker‐based (Figure S4a,b) mitochondrial labeling. NRF1, TFAM, and COXIV expression levels were significantly increased compared to SCR controls (Figure 1c). NRF1 mRNA transfection also stimulated mitochondrial biogenesis in a replicative senescence model, evidenced by significantly increased mitochondrial‐associated fluorescence (Figure S5a,b) and NRF1, TFAM, and COXIV expression (Figure S5c) in aged MSCs.

Mitochondrial biogenesis induction for therapeutic applications has been previously explored, with most strategies involving external stimuli (e.g., exercise) and their actions on the PGC‐1α pathway. As an example, calorie restriction‐induced PGC‐1α signaling resulted in proliferation of more efficient mitochondria capable of maintaining essential levels of ATP production (López‐Lluch et al., 2006). Melatonin treatment restored mitochondrial biogenesis in cardiomyocytes undergoing ischemia/reperfusion injury through activation of the 5′ adenosine monophosphate‐activated protein kinase (AMPK)/PGC‐1α pathway (Qi & Wang, 2020). Browning of white fat in response to cold exposure decreased the microRNA miR‐494‐3p, which in turn induced PGC‐1α and TFAM expression (Lemecha et al., 2018). Lastly, the transcriptional co‐activator with PDZ binding motif (TAZ) was recently identified as a novel mitochondrial biogenesis stimulator in skeletal muscle cells in response to exercise (Hwang et al., 2022). As the principal medium linking external signals to mitochondria, PGC‐1α responds to and is susceptible to a range of multiple, diverse stimuli that can impact its activity. Our goal was to induce mitochondrial biogenesis downstream of PGC‐1α through direct activation of NRF1. NRF1 regulates mitochondrial biogenesis through its influence on various mitochondrial proteins encoded by nuclear genes, including a significant number of proteins that constitute OXPHOS complexes (Scarpulla et al., 2012). Importantly, through its effects on TFAM gene expression, NRF1 consolidates nuclear control over mtDNA transcription and replication. Our findings demonstrate that upregulation of NRF1 expression effectively triggers mitochondrial biogenesis in MSCs, even under varied cell senescence scenarios.

### 2.2 NRF1 upregulation mitigated mitochondrial dysfunction in MSCs undergoing senescence

**FIGURE 2:**
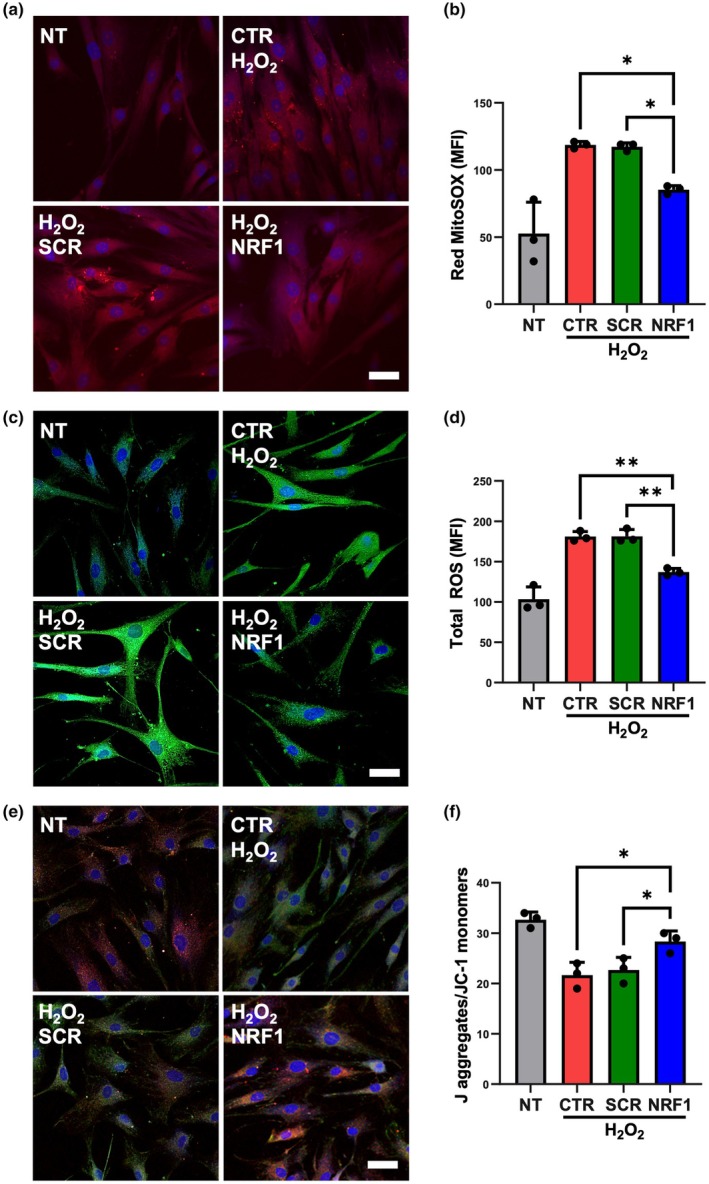
NRF1 overexpression reduced ROS and preserved mitochondrial health in MSCs exposed to oxidative stress. H_2_O_2_‐exposed (250 μM, 1 h) MSCs were transfected with either scrambled (SCR) or NRF1 mRNA. Controls consisted of non‐transfected MSCs (NT) and NT MSCs exposed to H_2_O_2_ (CTR). (a) Representative confocal microscopy images depicting MitoSOX stained MSCs. Oxidized MitoSOX reagent is represented in red and DAPI‐stained nuclei appear in blue. Scale bar = 50 μm. (b) Mean fluorescence intensity (MFI) of oxidized MitoSOX reagent quantified by flow cytometry. (c) Representative confocal microscopy images depicting intracellular total ROS via H2DCFDA staining. Oxidized H2DCFDA is represented in green and DAPI‐stained nuclei appear in blue. Scale bar = 50 μm. (d) MFI of oxidized H2DCFDA quantified by flow cytometry. (e) Representative confocal microscopy images of MSCs undergoing JC‐1 staining. JC‐1 monomers are represented in green, J aggregates in red, and DAPI‐stained nuclei in blue. Scale bar = 50 μm. (f) The ratio of the median fluorescence intensity (MedFI) of J aggregates to JC‐1 monomers quantified by flow cytometry. *p < 0.05; **p < 0.005.

During aging, high levels of ROS are produced alongside a decrease in antioxidant defenses (Maldonado et al., 2023). Cell senescence is closely associated with mitochondrial dysfunction, with senescent cells exhibiting reduced mitochondrial membrane potential (ΔΨm) and respiratory capacity per mitochondrion (Miwa et al., 2022). Compared to NT MSCs, MSCs exposed to H_2_O_2_ had increased mitochondrial ROS (mtROS) (Figure 2a,b) and intracellular total ROS levels (Figure 2c,d), and reduced ΔΨm (Figure 2e,f). Importantly, NRF1 mRNA transfection of H_2_O_2_‐exposed MSCs resulted in a decrease in mtROS and intracellular total ROS levels compared to H_2_O_2_‐exposed MSC SCR controls and protected against mitochondrial membrane depolarization. Similarly, MSCs undergoing replicative senescence displayed increased mtROS (Figure S6a,b) and intracellular total ROS levels (Figure S6c,d), as well as decreased ΔΨm (Figure S6e,f). NRF1 induction in MSCs undergoing replicative senescence also led to a decrease in mtROS and intracellular total ROS levels, with cells exhibiting lessened mitochondrial membrane depolarization.

**FIGURE 3:**
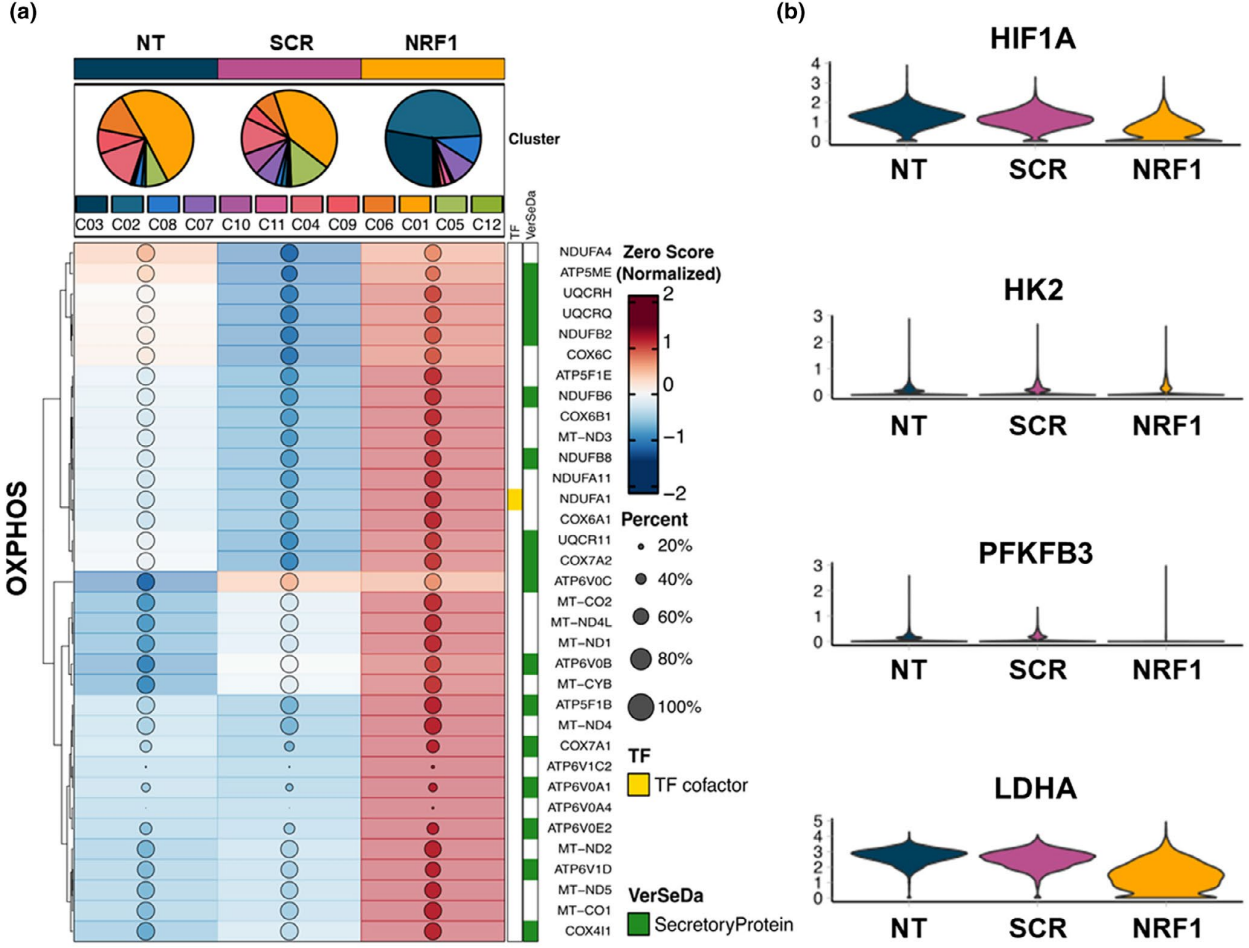
NRF1 priming potentiated mitochondrial bioenergetics in MSCs. MSCs were transfected with either scrambled (SCR) or NRF1 mRNA. Controls consisted of non‐transfected MSCs (NT). (a) Group heatmap illustrating the expression pattern of 34 genes associated with OXPHOS, as identified in the KEGG Oxidative phosphorylation pathway in control and SCR‐ or NRF1 mRNA‐transfected MSCs scRNA‐Seq dataset. Dot size indicates the percentage of cells expressing each marker and color represents normalized expression. Features were annotated using transcription factor (TF) database and vertebrate secretome database (VerSeDa). (b) Violin plots showing expression levels of glycolysis markers in control and SCR‐ or NRF1 mRNA‐transfected MSCs scRNA‐Seq dataset.

Oxidative stress and the ensuing mitochondrial membrane depolarization impair OXPHOS and ATP producing mechanisms (J. Park et al., 2011). A recent study involving RNA sequencing of bone marrow derived MSCs showed that aged rats had ~3000 differentially expressed genes, with downregulated genes involved in metabolic processes and mitochondria (Sun et al., 2022). Our goal was to reinforce mitochondrial function in MSCs to thwart cell senescence. To investigate the relationship between NRF1 activation and metabolic gene expression in MSCs, we studied two key metabolic pathways in our scRNA‐Seq datasets: glycolysis and OXPHOS. Expression of genes involved in OXPHOS was assessed using the KEGG oxidative phosphorylation reference pathway (HSA00190). A notable alteration in gene expression patterns of OXPHOS genes was observed in NRF1 primed MSCs compared to controls (Figure 3a). Specifically, 34 OXPHOS‐related transcripts demonstrated a significant increase in their expression levels within NRF1‐transfected MSCs compared to SCR mRNA treated MSCs. Conversely, analysis of glycolysis gene (HIF1A, HK2, PFKFB3, and LDHA) expression showed a significant decrease following NRF1 transfection (Figure 3b). This observation underscores a regulatory role of NRF1 in modulating the expression of genes associated with the balance of glycolysis and OXPHOS processes in MSCs, highlighting its impact on cellular metabolic pathways. Findings also signal that NRF1 activation can condition MSCs with a favorable metabolic programming capable of efficient bioenergetic handling, one that should enable MSCs to endure adverse pathological microenvironments characterized by oxidative stress.

**FIGURE 4:**
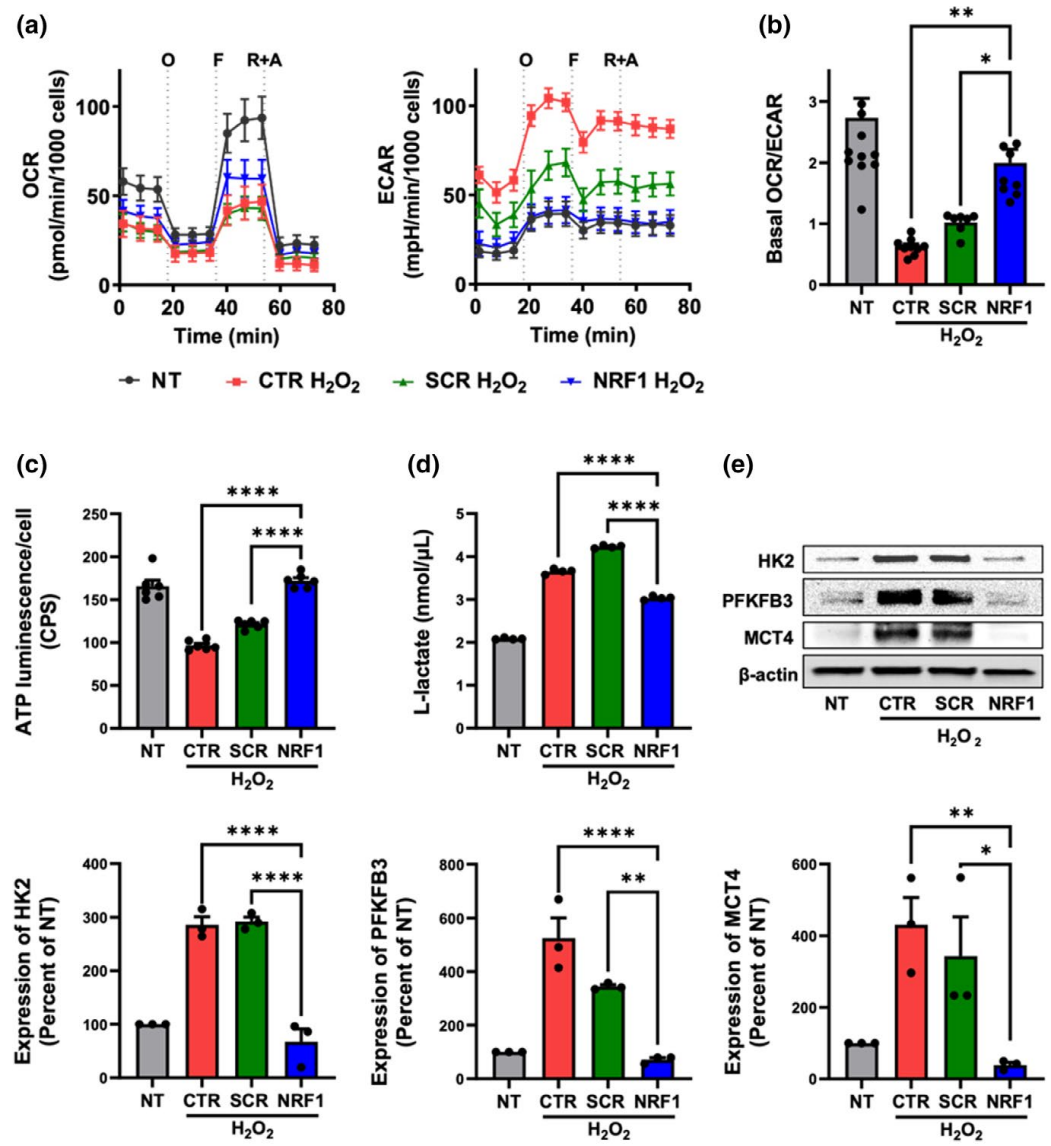
NRF1 overexpression enhanced OXPHOS and decreased glycolysis in MSCs exposed to oxidative stress. H_2_O_2_‐exposed (250 μM, 1 h) MSCs were transfected with either scrambled (SCR) or NRF1 mRNA. Controls consisted of non‐transfected MSCs (NT) and NT MSCs exposed to H_2_O_2_ (CTR). (a) Bioenergetic analysis of the effect of NRF1 overexpression in MSCs via examination of the oxygen consumption rate (OCR) and extracellular acidification rate (ECAR). O: oligomycin; F: FCCP; R + A: rotenone + antimycin A. (b) The ratio of basal OCR/ECAR of MSCs. (c) Relative intracellular ATP. (d) l‐lactate production. (e) Representative western blot of HK2, PFKFB3, and MCT4 expression in MSCs. Densitometric analysis was performed to quantify protein expression. Protein markers were normalized to β‐actin expression levels. *p < 0.05; **p < 0.005; ****p < 0.0001.

H_2_O_2_ had profound effects on MSC bioenergetics. Analysis of basal oxygen consumption rate (OCR) demonstrated that oxidative stress resulted in a decrease in basal respiration and maximal respiratory capacity in MSCs (Figure 4a). Extracellular acidification rate (ECAR) analysis after H_2_O_2_ exposure showed an increase in basal ECAR (Figure 4a), signaling a change toward a more glycolytic state. The basal OCR/ECAR ratio is an indicator of cellular bioenergetic balance and a gauge to determine how oxidative pathways and glycolysis are used for energy production. Our results show that the basal OCR/ECAR ratio was significantly decreased following H_2_O_2_ exposure of MSCs (Figure 4b). Bioenergetic changes involving OXPHOS and glycolysis were reflected in ATP production, and a significant decrease in intracellular ATP was observed in MSCs treated with H_2_O_2_ (Figure 4c). NRF1 mRNA transfection of H_2_O_2_‐exposed MSCs impacted cell respiration by increasing basal and maximal OCR (Figure 4a). A decrease in basal ECAR translated to an increase in the basal OCR/ECAR ratio (Figure 4b). NRF1 induction in H_2_O_2_‐exposed MSCs also led to a significant increase in intracellular ATP (Figure 4c), indicative of an increase in OXPHOS and cell respiration. Senescent cells have previously been shown to adopt features associated with enhanced glycolysis (Wiley & Campisi, 2016). Our findings show an increase in the levels of l‐lactate, the end product of glycolysis, in the supernatant of H_2_O_2_‐exposed MSCs, with NRF1 overexpression resulting in reduced l‐lactate levels in the supernatant (Figure 4d). Tanner et al. showed that increased glycolytic flux is controlled by glucose import, hexokinase, phosphofructokinase, and lactate export (Tanner et al., 2018). NRF1 overexpression in MSCs undergoing H_2_O_2_ exposure led to a significant reduction in the expression of hexokinase 2 (HK2), 6‐phosphofructo‐2‐kinase/fructose‐2,6‐bisphosphatase 3 (PFKFB3), and monocarboxylate transporter 4 (MCT4), the latter responsible for lactate efflux, compared to H_2_O_2_‐exposed controls (Figure 4e). Like the stress‐induced premature senescence model, NRF1 induction had profound impacts on cell bioenergetics and glycolysis in a replicative model of cell senescence. Compared to aged controls, NRF1 induction in cells undergoing replicative senescence had an increased basal OCR/ECAR ratio (Figure S7a,b) and ATP production (Figure S7c). Moreover, NRF1 overexpression in aged cells reduced replicative senescence‐associated glycolysis, as evidenced by reduced levels of l‐lactate in the supernatant (Figure S7d) and glycolytic marker expression (Figure S7e). Our findings demonstrate that NRF1 induction in MSCs undergoing senescence resulted in metabolic potentiation evidenced by enhanced OXPHOS and ATP production, as well as decreased glycolysis.

**FIGURE 5:**
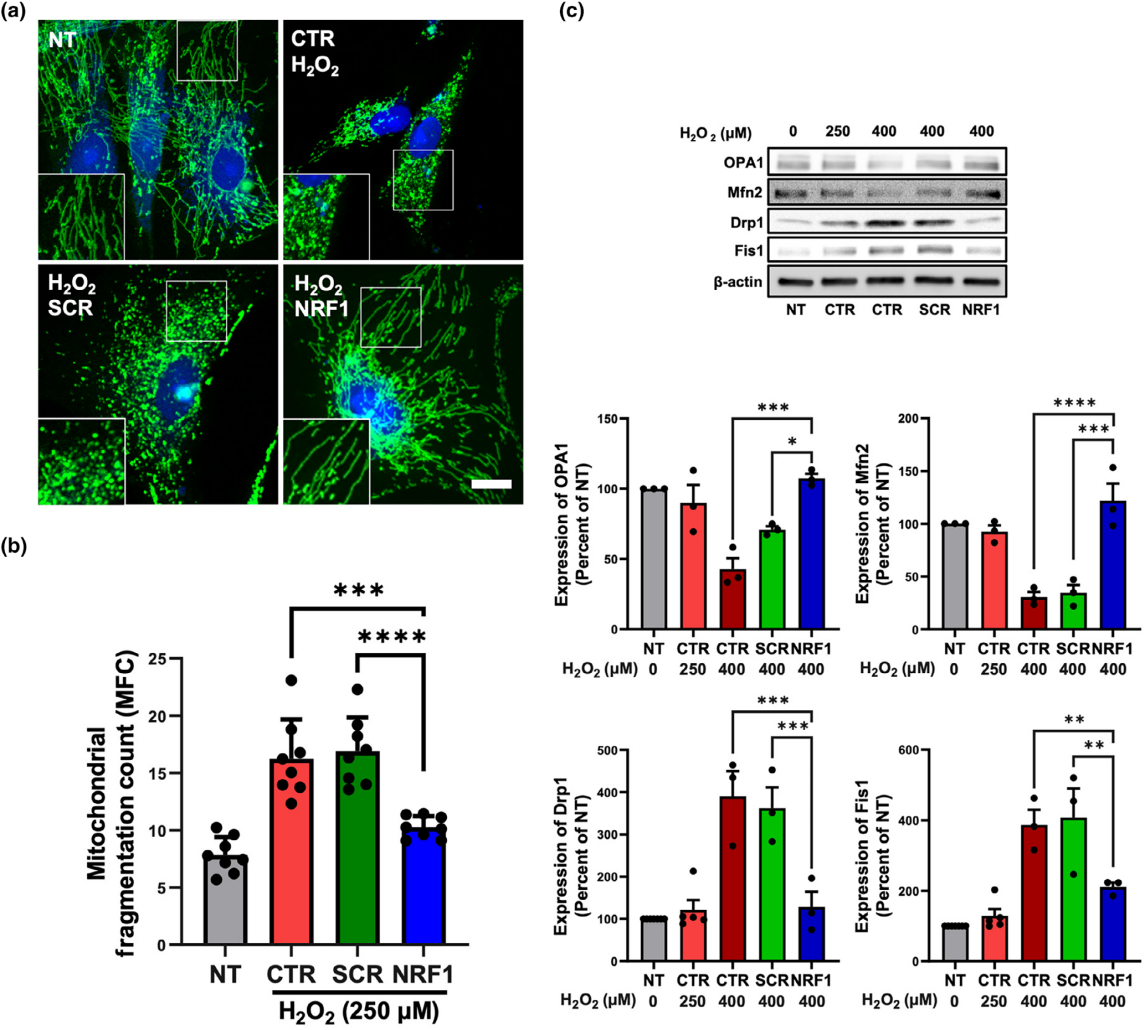
NRF1 overexpression preserved mitochondrial dynamics in MSCs exposed to oxidative stress. (a) Representative confocal microscopy images of AcGFP1‐expressing mitochondrial morphology in MSCs. H_2_O_2_‐exposed (250 μM, 1 h) MSCs were transfected with either scrambled (SCR) or NRF1 mRNA. Controls consisted of non‐transfected MSCs (NT) and NT MSCs exposed to H_2_O_2_ (CTR). Green represents AcGFP1‐associated fluorescence and blue represents DAPI nuclear staining. Scale bar = 15 μm. (b) Mitochondrial fragmentation count (MFC). (c) Representative western blot analysis of mitochondrial dynamics‐related proteins (OPA1, Mfn2, Drp1, and Fis1) in MSCs. MSCs were exposed to H_2_O_2_ (250 μM or 400 μM, 1 h) and subsequently transfected with either SCR or NRF1 mRNA. Controls consisted of non‐transfected MSCs (NT) and NT MSCs exposed to H_2_O_2_ (CTR). Densitometric analysis was performed to quantify protein expression, normalized to β‐actin levels. *p < 0.05; **p < 0.005; ***p < 0.0005; ****p < 0.0001.

Mitochondria are highly dynamic organelles that exist in a varied range of morphologies necessary for mitochondrial homeostasis. These include fragmented states if undergoing fission, and continuous network structures if undergoing fusion, the latter state associated with increased energy production, apoptotic stress protection, and increased cell proliferation (Hoitzing et al., 2015). In NT MSCs, mitochondria appeared in elongated and networked structures (Figure 5a). Rather than displaying fused networks, mitochondria in MSCs treated with H_2_O_2_ existed in discrete fragmented states (Figure 5a), a morphological feature associated with mitochondrial fission (Durand et al., 2019). In contrast, NRF1‐transfected MSCs exposed to H_2_O_2_ had a continuous reticula mitochondrial structure like NT MSCs (Figure 5a) and showed less fragmentation (Figure 5b). Impaired mitochondrial fusion and fission upsets the healthy pool of mitochondria, and aberrant mitochondrial homeostasis is manifested by imbalances in the expression of fusion and fission proteins. In fusion, optic atrophy 1 (OPA1) and mitofusins (Mfn1 and Mfn2) bind the inner and outer mitochondrial membrane, respectively, of two adjacent mitochondria (Liu, McIntyre, et al., 2020). With regards to fission, mitochondrial fission 1 protein (Fis1) is responsible for dynamin‐related protein 1 (Drp1) recruitment and assembly on mitochondrial constriction sites, which in turn oligomerizes and severs the mitochondrion through scission (Chen et al., 2023). Mfn2 loss (Li et al., 2024) and age‐related declines in OPA1 (Tezze et al., 2017) have been shown to induce a senescence phenotype. Upregulation of Drp1 expression was also shown to lead to senescence (You et al., 2023). Despite evidence of mitochondrial fragmentation in MSCs exposed to H_2_O_2_ for 1 h at a dose of 250 µM, no significant alterations in expression levels of fusion and fission proteins were observed following this H_2_O_2_ exposure regimen (Figure 5c). This result was principally due to the concentration of H_2_O_2_ (250 μM) used in our studies, which was in line with findings from previous studies (Garcia et al., 2018; Iqbal & Hood, 2014). At an H_2_O_2_ dose of 400 μM, imbalances of mitochondrial fission and fusion were observed in MSCs, specifically, increased fission and decreased fusion (Figure 5c). Importantly, NRF1 induction in MSCs exposed to H_2_O_2_ at a dose of 400 μM maintained balanced mitochondrial dynamics. In the replicative model of senescence, increased mitochondrial fragmentation was observed (Figure S8a,b), accompanied by a clear imbalance in the expression of fusion and fission proteins (Figure S8c). As in the stress‐induced premature senescence model, NRF1 induction in aged MSCs resulted in decreased mitochondrial fragmentation and balanced mitochondrial dynamics of fusion and fission (Figure S8c). Taken together, NRF1 overexpression in MSCs undergoing senescence preserved mitochondrial homeostasis.

The overarching function of mitochondrial biogenesis is to maintain mitochondrial quality and ensure adequate ATP production. Findings indicate that NRF1 induction reduced intracellular levels of ROS and improved mitochondrial health and respiration. Our laboratory previously demonstrated that boosting mitochondrial content via transplantation of exogenous mitochondria was associated with a transition from a glycolytic phenotype to enhanced OXPHOS and increased cellular energy production (Baudo et al., 2023; Liu et al., 2022; Wu et al., 2018). Similar effects on cell bioenergetics due to increased mitochondrial mass have been demonstrated by other groups. Adenoviral‐mediated induction of PGC‐1α expression in primary human fetal retinal pigment epithelial cells and an ARPE‐19 cell line increased the expression of OXPHOS, fatty acid β‐oxidation, and antioxidant genes, ultimately resulting in induction of mitochondrial respiration, fatty acid oxidation, and antioxidant capacity (Iacovelli et al., 2016). Specific to NRF1, Zhao et al. demonstrated that miR‐504 was capable of directly targeting NRF1, resulting in decreased expression of OXPHOS complexes I, III, and IV, highlighting the profound impact that microRNA‐mediated downregulation of NRF1 has on mitochondrial metabolism and respiratory function (Zhao et al., 2015). Recently, NRF1 gain‐of‐function resulted in an antioxidant response involving the activation of ROS scavengers, which when combined with increased proteasomal activity, guarded against ischemia/reperfusion injury in mouse hearts (Cui et al., 2021). Mitochondrial biogenesis was also associated with reduced oxidative stress in the aforementioned caloric restriction study (López‐Lluch et al., 2006), while melatonin‐induced mitochondrial biogenesis also led to an inhibition of oxidative stress and enhanced mitochondrial metabolism (Qi & Wang, 2020). These studies highlight the potential of NRF1 activation as a strategy to protect against oxidative stress and mitochondrial dysfunction, and to bioenergetically potentiate cells.

### 2.3 NRF1 induction abrogated senescence in MSCs

MSC senescence resulting from either oxidative stress or expansion to obtain clinically adequate cell numbers proves detrimental to the reparative potential of MSC‐based treatment strategies by limiting their replicative capacity. The differentiation bias is also altered in senescent MSCs, displaying an increased propensity to differentiate into adipocytes compared to osteoblasts (Laschober et al., 2011). Moreover, senescence affects MSC immunomodulation and paracrine signaling. Cells undergoing senescence can influence their microenvironment through increased secretion of senescence‐associated secretory phenotype (SASP) factors consisting of cytokines and growth factors that contribute to inflammation through effects such as immune cell recruitment (Rodier & Campisi, 2011). Thus, senescent MSCs can instead fulfill degenerative roles that drive disease progression. Moreover, MSCs undergoing senescence alter their extracellular vesicle (EV) composition, particularly with regards to composition of miRNAs (Liu & Chen, 2020). Senescent, late passage MSCs were found to secrete smaller sized EVs than early passage MSCs and had high levels of expression of miR‐146a‐5p, a potential senescence‐associated marker, highlighting the contribution of senescent MSCs to age‐related disease progression (e.g., Alzheimer's disease) (Lei et al., 2017).

**FIGURE 6:**
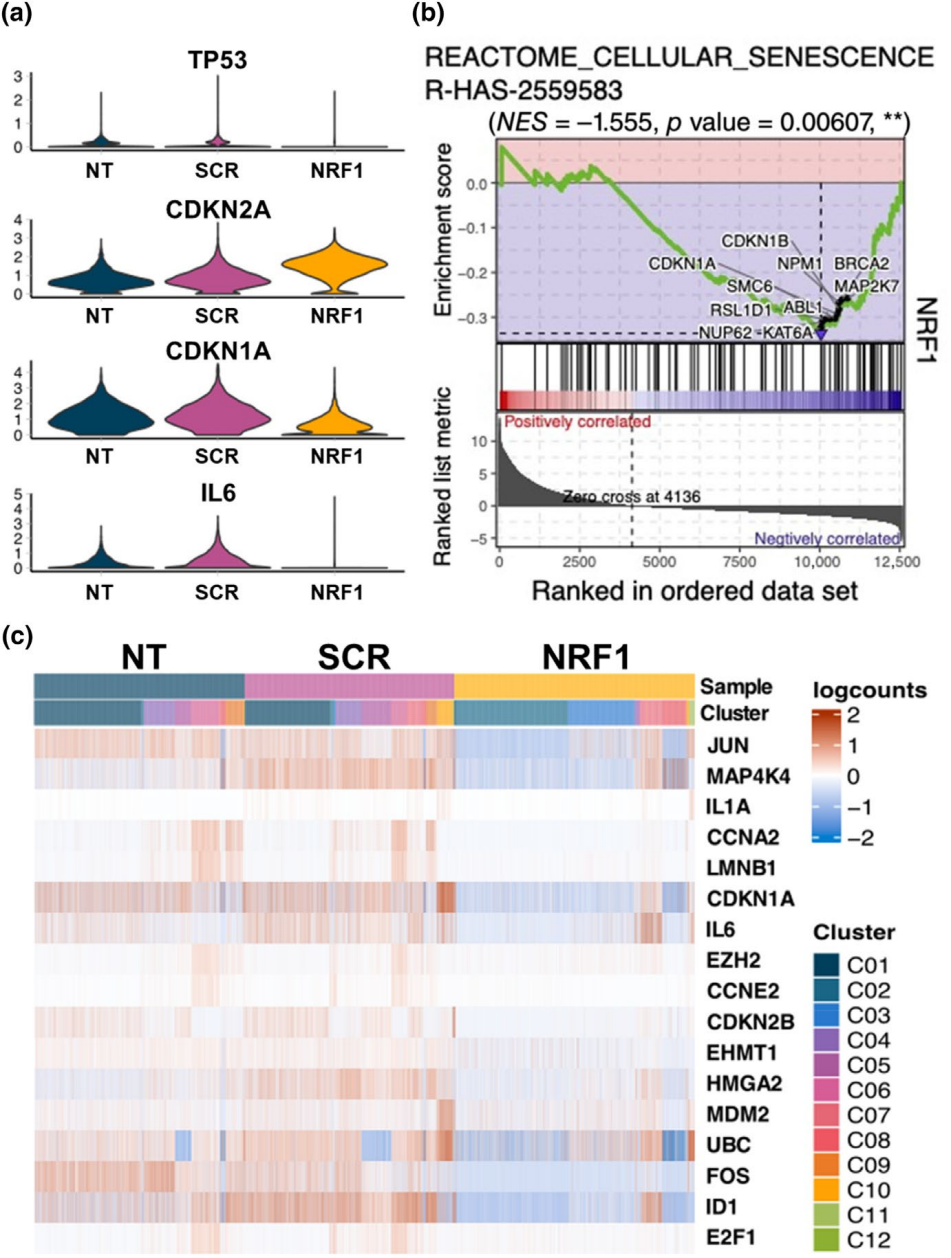
NRF1 priming downregulated senescence processes in MSCs. MSCs were transfected with either scrambled (SCR) or NRF1 mRNA. Controls consisted of non‐transfected MSCs (NT). (a) Violin plots showing expression levels of typical senescence markers in control (NT) and SCR‐ or NRF1 mRNA‐transfected MSCs scRNA‐Seq datasets. (b) Gene set enrichment analysis (GSEA) enrichment plots showing suppression of senescence related pathways in NRF1 mRNA‐transfected MSCs compared to SCR control. (c) Heatmap illustrating the expression pattern of 17 genes at different clusters associated with senescence in MSCs, as identified in the Cellular Senescence pathway from the Reactome Pathways dataset.

Given the detrimental effects of senescence on MSC function, as well as the potential to aggravate disease progression, strategies aimed at abrogating senescence stand to enhance therapeutic outcomes of MSC‐based approaches. We hypothesized that NRF1 induction would offset cell senescence. Our scRNA‐Seq dataset revealed that, apart from CDKN2A expression, a significant downregulation of TP53, CDKN1A, and IL6 was observed following NRF1 transfection (Figure 6a). Utilizing gene set enrichment analysis (GSEA), specifically GOBP_CELLULAR_SENESCENCE (GO:0090398) and REACTOME_CELLULAR_SENESCENCE (R‐HAS‐2559583) gene sets, we observed a consistent downregulation of genes associated with senescence (Figure 6b). Lastly, NRF1 exhibited a significant inhibitory effect on the expression of 17 senescence‐related genes (Figure 6c). These findings are indicative of the potential of NRF1 priming to robustly deter the development of senescence in MSCs.

**FIGURE 7:**
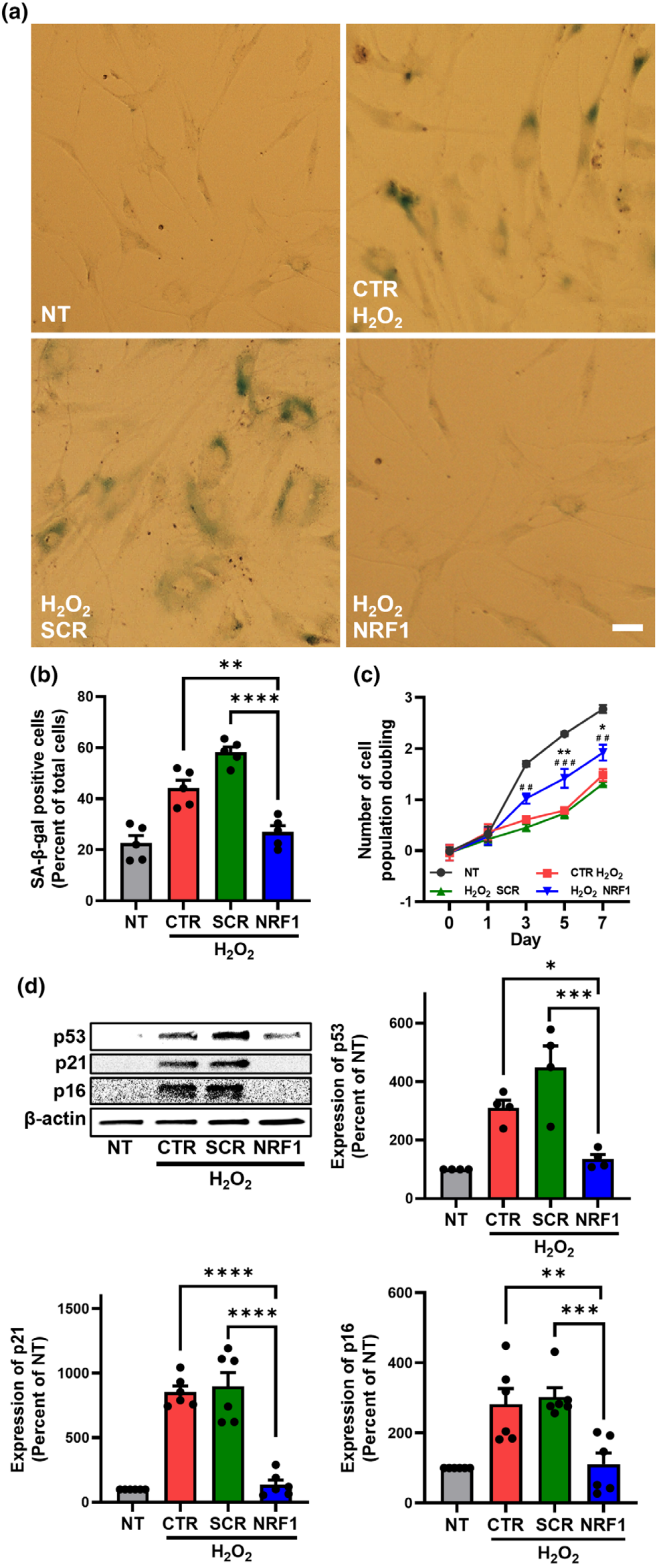
NRF1 overexpression in MSCs suppressed oxidative stress‐induced senescence. H_2_O_2_‐exposed (250 μM, 1 h) MSCs were transfected with either scrambled (SCR) or NRF1 mRNA. Controls consisted of non‐transfected MSCs (NT) and NT MSCs exposed to H_2_O_2_ (CTR). (a) Representative light microscopy images of SA‐β‐gal activity in MSCs. Scale bar = 25 μm. (b) SA‐β‐gal activity expressed as the percentage of SA‐β‐gal positive cells. (c) Number of cell population doubling (NCPD) as a function of time. (d) Representative western blot of p53, p21, and p16 expression in MSCs. Densitometric analysis was performed to quantify protein expression. Protein markers were normalized to β‐actin expression levels. *p < 0.05; **p < 0.005; ***p < 0.0005 ****p < 0.0001. For NCPD, two‐way ANOVA followed by Tukey's multiple comparison test was used for statistical analysis: *p < 0.05; **p < 0.005 versus CTR H_2_O_2_ group; ^##^ *p* < 0.005; ^###^ *p* < 0.0005 versus H_2_O_2_+SCR group.

NRF1 induction led to a significant attenuation of stress‐induced premature senescence in MSCs (Figure 7). Expression of SA‐β‐gal, a marker for senescence and cell aging, increased in MSCs exposed to H_2_O_2_ (Figure 7a,b). In contrast, MSCs exposed to H_2_O_2_ and transfected with NRF1 mRNA had SA‐β‐gal activity levels comparable to those of NT MSCs. A well‐known hallmark of cell senescence is cessation of cell proliferation, and herein, oxidative stress reduced the population doubling time of MSCs (Figure 7c). NRF1 overexpression in MSCs rescued H_2_O_2_‐induced effects on cell proliferation. Activation of the p53/p21^WAF1/CIP1^ and p16^INK4A^/pRB pathways play an important role in senescence regulation, and oxidative stress has been shown to upregulate p16 and p53 (Weng et al., 2022). The senescence‐associated markers p53, p21, and p16 were found elevated in MSCs exposed to H_2_O_2_ compared to NT MSCs (Figure 7d). Contrastingly, NRF1 transfection of MSCs exposed to H_2_O_2_ resulted in reduced levels of p53, p21, and p16.

Similar results were observed in a replicative model of senescence, with aged cells displaying increased SA‐β‐gal activity levels and heightened expression of senescence‐associated markers (Figure S9). Importantly, NRF1 induction in cells undergoing replicative senescence reduced SA‐β‐gal activity (Figure S9a,b) and p53, p21, and p16 expression (Figure S9c). In both the stress‐induced premature senescence and replicative senescence models, the effects of NRF1 mRNA transfection on senescent MSCs were compared to those of ABT263, a potent and selective senolytic agent (Chang et al., 2016). ABT263 resulted in decreased SA‐β‐gal activity levels and expression of senescence‐associated markers compared to senescence controls (Figures S9 and S10), reaffirming that NRF1 overexpression was capable of abrogating senescence. Of note, ABT263 exerted distinct effects on stress‐induced premature senescence compared to replicative senescence, particularly regarding the inhibition of p53 expression. While ABT263 resulted in an inhibition of p21 and p16 expression in both models, significant inhibition of p53 expression was observed in the stress‐induced premature senescence model, but not in the replicative senescence. This is possibly due to MSCs in the stress‐induced premature senescence model having not yet fully transitioned into a senescent state, causing them to respond similarly to younger cells at the time of treatment, and it is also likely that apoptosis was selectively induced via a p53‐independent pathway (Seluanov et al., 2001).

Our findings show that NRF1 mRNA transfection of MSCs undergoing senescence improved mitochondrial function and reduced senescence. We next compared NRF1 induction to bezafibrate, an enhancer of PGC‐1α levels (Augustyniak et al., 2019), and doxycycline, known to reduce mitochondrial encoded proteins and impair mitochondrial respiration and membrane potential (Dijk et al., 2020). Bezafibrate indeed increased mitochondrial content in MSCs, while doxycycline reduced mitochondrial mtDNA levels and impacted mitochondrial function (Figures S11). Both NRF1 induction and bezafibrate reduced MSC senescence in both models, evidenced by decreased SA‐β‐gal activity and lower expression of senescence markers p53, p21, and p16 (Figures S12 and S13). Doxycycline had minimal impact on SA‐β‐gal activity compared to senescence controls, and showed different effects on the expression of p53, p21, and p16 between the H_2_O_2_‐induced and replicative senescence models, likely due to differences in senescence mechanisms and timing. In the stress‐induced premature senescence model, the antioxidant properties of doxycycline (Clemens et al., 2018) likely reduced ROS, decreasing DNA damage and attenuating the upregulation of p21 and p16 without significantly affecting p53 levels within 24 h. This suggests that doxycycline may delay senescence progression by mitigating oxidative stress. Studies indicate that p53 activation occurs rapidly after DNA damage, while p21 and p16 increase later (within 12–24 h), highlighting a temporal sequence in the senescence response (Johmura et al., 2014). Conversely, replicative senescence involves cumulative telomere shortening and sustained activation of p53 and p21 over extended cell division—five passages before treatment in our study. Doxycycline showed minimal efficacy in this model, slightly reducing p53 levels but having less impact on p21 and p16 expression compared to NRF1 induction or bezafibrate treatment. This is likely because its antioxidant action cannot reverse established telomere‐driven senescence. Our results indicate that induction of mitochondrial biogenesis is associated with senescence hindrance. However, it is important to note that NRF1 exerts effects on other cell processes apart from mitochondrial biogenesis, with Hu et al. recently showing that NRF1 acts as a potent integrator of redox regulation and a regulator of antioxidant genes (Hu et al., 2022). Our findings demonstrate that NRF1 induction in MSCs undergoing stress‐induced premature senescence and replicative senescence resulted in an increase in the expression of the antioxidant proteins GSR, SOD1, and TXN1 (Figures S14 and S15, respectively). Thus, the effects of NRF1 induction on senescence may very well be the result of a broader maintenance of proper functioning of mitochondrial processes that extend beyond mitochondrial biogenesis.

Senescence is a major obstacle to MSC therapies, limiting their proliferative capacity and differentiation potential, and reconfiguring their secretome toward a more injurious phenotype that can benefit disease progression. Thus, strategies aimed at senescence deterrence remain a clinically relevant unmet need and an active area of investigation, with several approaches principally involving proliferation induction in MSCs. Coutu et al. demonstrated that MSC senescence could be inhibited through PI3K/AKT‐MDM2‐mediated FGFR1/2 signaling that promotes MSC proliferation (Coutu et al., 2011). Similarly, the Notch signaling pathway was identified as an important regulator of MSC proliferation, and activation of the pathway via Notch ligand Jagged1 (JAG1) inhibited MSC senescence (Tian et al., 2017). miR‐34a, a tumor suppressor microRNA, is overexpressed in aged cells and leads to senescence through its actions on Sirtuin 1 (SIRT1), which reduces MSC proliferation (Park et al., 2015). Mokhberian et al. showed that miR‐34a inhibition resulted in enhanced proliferation and reduced senescence in MSCs (Mokhberian et al., 2020). Lastly, cell aging is associated with a dysregulation of lysosomal pH, and promotion of lysosomal acidification via the small molecule 3‐butyl‐1‐chloro imidazo [1, 5‐a] pyridine‐7‐carboxylic acid (SGJ) resulted in decreased senescence through upregulation of lysosome‐associated membrane protein 1 (LAMP1) and 2 (LAMP2) expression (Wang et al., 2018).

To the best of our knowledge, this study represents the first report of gene therapy for the direct activation of NRF1 to increase mitochondrial mass and stave off senescence through metabolic potentiation. Our findings demonstrate that NRF1 induction increased mitochondrial content, which correlated with improved mitochondrial function and dampened senescence (Figure S16). The ability of NRF1 induction to offset stress‐induced senescence may prove beneficial in oxidative in vivo microenvironments. Similar results in a replicative senescence model can be extrapolated toward the benefit of autologous MSC therapies involving MSCs isolated from older donors, whose cells have been exposed to in vivo intrinsic aging (Baker et al., 2015). Targeting mitochondrial dysfunction to hinder MSC senescence has been previously explored. A mitochondrial antioxidant, Mito‐TEMPO, was able to restore mitochondrial function and reverse senescence in MSCs exposed to simulated microgravity (Lv et al., 2023). In a study by Yang et al., ascorbic acid inhibited ROS production and senescence in aging MSCs that underwent d‐galactose‐induced glycolytic programming (Yang, Teng, et al., 2018). Recently, mitochondrial biogenesis strategies have emerged as approaches to improve stem cell health. Rg3 isolated from *Panax ginseng* was shown to increase MSC proliferation and reduce senescence through a mechanism involving cytosolic Ca^2+^ concentration elevation to upregulate mitochondrial biogenesis and antioxidant enzymes (Hong et al., 2020). Taken together, reinforcing proper mitochondrial function in MSCs can offset senescence processes, which in turn should translate to therapeutic enhancement of MSC‐based transplantation strategies.

## 3 CONCLUSIONS

MSCs represent the archetype cell therapy, having shown immense potential for regenerative medicine approaches. However, senescence represents a major limitation for the clinical translation of MSCs. In this study, we demonstrated that NRF1 overexpression boosted mitochondrial biogenesis in MSCs, enhancing metabolic function and preserving mitochondrial health even under oxidative stress conditions. By reinforcing mitochondrial function, NRF1 induction effectively counteracted senescence processes in both stress‐induced premature senescence and replicative senescence models. These findings suggest that promoting mitochondrial biogenesis through NRF1 overexpression is a powerful strategy to enhance MSC robustness and longevity. By mitigating senescence, NRF1 induction in MSCs could potentially exhibit improved therapeutic outcomes when transplanted into adverse pathological environments characterized by oxidative stress. Future work will involve continued exploration of NRF1 effects on MSC processes such as immunomodulation and differentiation. Work will also focus on the exploration of metabolically primed MSCs in in vivo models of disease, as well as additional MSC engineering to heighten MSC long‐term fitness and efficacy. Overall, our work highlights the critical role of mitochondrial biogenesis in maintaining MSC function and underscores the potential of NRF1 overexpression as a translational strategy to overcome senescence‐related limitations in MSC‐based therapies.

## 4 MATERIALS AND METHODS

### 4.1 Materials

Human NRF1 mRNA was purchased from Trilink Biotechnologies (San Diego, CA). Scrambled (SCR) mRNA was purchased from the Houston Methodist RNAcore (Houston, TX). Lipofectamine™ Messengermax™ reagent was purchased from ThermoFisher Scientific (Waltham, MA). The lentiviral particle transduction system for introduction of AcGFP1 in mitochondria was purchased from Takara Bio (San Jose, CA). Protamine sulfate was purchased from ThermoFisher Scientific. Puromycin was purchased from MilliporeSigma (Burlington, MA). Bezafibrate was purchased from ThermoFisher Scientific, doxycycline was purchased from Sigma‐Aldrich (St. Louis, MO), and ABT263 (Navitoclax) was purchased from APExBio Technology LLC (Houston, TX). Bone marrow‐derived mesenchymal stem cells (MSCs) were purchased from Lonza (Durham, NC) and resuspended in MSC basal media (Lonza) supplemented with MSCGM™SingleQuots™ kit (Lonza). Cells were expanded per the manufacturer's recommendation until reaching 80% confluency in a humidified incubator with 5% CO_2_ at 37°C. Unless otherwise specified, all other chemicals and reagents were obtained from Sigma‐Aldrich.

### 4.2 Cell seeding, H_2_O_2_ exposure, induction of replicative senescence, and mRNA transfection

For all experiments except MSC proliferation, MSCs were seeded at a density of 0.8 × 10^4^ cells/cm^2^. After 24 h, MSCs underwent preincubation with 250 μM of H_2_O_2_ for 1 h, a concentration and treatment time that proved non‐lethal in MSCs (Figure S17). For assessment of mitochondrial dynamics markers by western blot in the stress‐induced premature senescence model, MSCs also underwent preincubation with 400 μM of H_2_O_2_ for 1 h. In all experiments involving MSCs undergoing replicative senescence, cells were cultured up to passage 9 and subsequently seeded, with passage 10 (p10) being designated for use in experiments. To ensure consistency in cell culture conditions, cells were counted and seeded at a density of 0.5 × 10^4^ cells/cm^2^ at each passage. MSCs were grown to confluence before being utilized in experimental procedures. Subsequently, MSCs were washed with PBS and starved with MSC basal medium containing 0.2% FBS for 6 h. Transfection was then carried out using 1 μg/mL of either NRF1 mRNA or SCR mRNA in Lipofectamine™ MessengermaxTM reagent. After 6 h, cells were washed with PBS and replenished with complete MSC medium for an additional 18 h, at which point analyses were performed. All experiments had non‐transfected (NT) MSCs as controls. Throughout the study, NT MSCs exposed to H_2_O_2_ (CTR) also served as controls. In the replicative senescence model, controls consisted of non‐transfected MSCs at passages 5 (p5) and 10 (p10). As controls in senescence studies, MSCs were incubated with bezafibrate (50 μM), ABT263 (1 μM), or doxycycline (10 μg/mL) for 24 h. Drugs were resuspended in fresh MSC medium before administration. In the case of the stress‐induced premature senescence mode, bezafibrate, ABT263, or doxycycline were added 1 h after H_2_O_2_ incubation, following H_2_O_2_ wash out.

### 4.3 Lentiviral transduction

A lentiviral particle transduction system for introduction of the AcGFP1 protein for specific expression in mitochondria (mitoAcGFP1) was used to selectively label mitochondria in MSCs (Figure S18a). Briefly, MSCs were seeded at a density of 0.8 × 10^4^ cells/cm^2^, and 24 h later, complete medium containing lentiviral particles were applied to the MSCs at a multiplicity of infection (MOI) of 10. Protamine sulfate stock solution was then directly added, and after an additional 24 h, the medium was replaced with fresh complete MSC medium and cells cultured for an additional 48 h. Subsequently, cells were subjected to selection with puromycin for an additional 48 h, resulting in mitoAcGFP1‐expressing MSCs. Expression of AcGFP1 in MSCs (Figure S18b) was visually examined using an EVOS cell imaging system (ThermoFisher Scientific).

### 4.4 Single‐cell RNA sequencing

Single‐cell RNA sequencing (scRNA‐Seq) of NRF1 or SCR mRNA treated healthy MSCs was performed by EMPIRI Inc. (Houston, TX) with NT MSCs serving as controls. MSCs were converted into a single‐cell suspension before capture via the 10× Chromium Controller. MSC samples with >95% viability were loaded into ChipK to target 5000 cells per group. The generation of single‐cell cDNA libraries utilized the 5′ transcriptomics V2 kit from 10× Genomics in accordance with the manufacturer's protocol.

### 4.5 scRNA‐Seq analysis

Raw sequencing reads were aligned to the GRCh38‐2020‐A human genome and genes were quantified as UMI counts using Cell Ranger (v5; RRID:SCR_017344) software from 10X Genomics, following the default parameters. Subsequently, doublets were removed using DoubletFinder (v2.0.3; RRID:SCR_018771), with an expected doublet rate of 7.5% based on Poisson statistics. Downstream analysis utilized filtered feature counts generated by Cell Ranger. Low‐quality single cells were identified using the RunMiQC function from the SeuratWrappers package (v0.3.19; RRID:SCR_022555) and subsequently removed. After this quality control step, single cells were normalized and clustered using Seurat (Stuart et al., 2019) (v5.0.1; RRID:SCR_016341) (Figure S19). Gene expression counts at the single‐cell level were normalized to the library size and log2‐transformed. PCA reduction was performed using Seurat (Stuart et al., 2019), focusing on the top 2000 most variable genes in the dataset. To account for variations between samples, computed PCAs underwent batch correction using Harmony (v1.1.0; RRID:SCR_018809). These batch‐corrected PCs (at a ratio of 1:40) served as input for Louvain‐based graphing, with a resolution of 0.5. Cluster and sample‐specific marker genes were identified using the fast Wilcoxon rank‐sum test wilcoxauc function from the presto R package (V1.0.0; github.com/immunogenomics/presto). Finally, results were visualized using ggplot2 (v3.3.3; RRID:SCR_014601), SCP (v0.5.6; github.com/zhanghao‐njmu/SCP), and ComplexHeatmap (v2.16.0; RRID:SCR_017270) packages in R.

### 4.6 Gene set enrichment analysis

We employed the fGSEA (v1.26.0; RRID:SCR_020938) R package to assess the enrichment of Reactome, KEGG, Biocarta, and Hallmark gene sets obtained from MsigDB (RRID:SCR_016863, msigdbr R package v7.5.1). As input, we utilized pre‐ranked gene lists generated through a fast Wilcoxon rank‐sum test (scRNA‐Seq) from the presto R package (v1.0.0; github.com/immunogenomics/presto). Finally, GSEA enrichment plots were created using SCP (v0.5.6; github.com/zhanghao‐njmu/SCP).

### 4.7 Reverse transcription‐quantitative PCR

NRF1 mRNA expression was examined by reverse transcription‐quantitative PCR (RT‐qPCR). Briefly, MSCs were seeded and after 24 h, cells were washed twice with pre‐warmed PBS and starved in a serum‐deprived medium containing 0.2% FBS in MSC basal medium. Transfection was performed as previously described and mRNA harvested after 24, 48, and 72 h. Post‐transfection, cells were washed twice, and RNA isolated using TRIzol, followed by genomic DNA digestion with DNase I. A total of 0.8 μg of RNA was used for reverse transcription (R5600‐505, GeneDepot), and RT‐qPCR was performed using a QuantStudio 7 Pro thermal cycler (ThermoFisher), following the manufacturer's guidelines for SYBR master mix (Q5602‐005, GeneDepot). RT‐qPCR primers used were as follows: 5′‐GGCAACAGTAGCCACATTGGCT‐3′ and 3′‐GTCGTCTGGATGGTCATCTCAC‐5′ for hNRF1 (purchased from Origene, Rockville, MD); and 5′‐TGGCAAATTCCATGGCACCGT‐3′ and 3′‐CCCATGACGAACATGGGGGC‐5′ for hGAPDH (purchased from Sigma‐Aldrich). NRF1 mRNA expression was normalized to GAPDH expression, and fold changes were calculated using the ^ΔΔ^Cq method.

### 4.8 Intracellular mitochondria visualization and quantification

Mitochondria were visualized by either mitoAcGFP1 labeling or MitoTracker staining. To visualize mitochondrial contents in mitochondria biogenesis and fragmentation experiments in the stress‐induced premature senescence model, mitoAcGFP1‐expressing MSCs were used. MitoTracker™ Green FM (Invitrogen) was used to counterstain mitochondria in MSCs undergoing stress‐induced premature senescence and replicative senescence in experiments involving visualization of effects of NRF1 induction on mitochondrial biogenesis. To visualize mitochondrial fragmentation in MSCs undergoing replicative senescence, MitoTracker™ Deep Red FM (Invitrogen) was employed as a counterstain. MitoTracker™ Deep Red FM was also used in experiments involving time course analysis of the effect of NRF1 overexpression, as well as bezafibrate and doxycycline treatments, on mitochondrial content. For all confocal experiments, MSCs were seeded in NuncTM Lab‐Tek™ II chamber slides (ThermoFisher Scientific). Following mRNA transfection, cells were washed with PBS, fixed with 4% paraformaldehyde (PFA), and permeabilized using a 0.2% Triton X‐100/PBS solution for 5 min. Afterward, cells were blocked with a 2% FBS/PBS solution for 1 h. Following incubation with Alexa Fluor™ 555 Phalloidin or Alexa Fluor™ 488 Phalloidin (ThermoFisher Scientific) for 1 h and DAPI for 15 min, fluorescence images were captured using the Nikon A1 Confocal Imaging System.

To assess mitochondrial mass after mRNA transfection, AcGFP1‐expressing MSCs or MitoTracker™ Green FM‐stained MSCs were washed with PBS, trypsinized, and the mean fluorescence intensity of AcGFP1 or MitoTracker™ Green FM quantified using an LSRII Flow Cytometer (BD Biosciences, Franklin Lakes, NJ).

### 4.9 Western blot analysis

Protein expression was evaluated using Western blot analysis. Prior to cell lysis, cells were treated with a protease and phosphatase inhibitor cocktail (GenDEPOT, Houston, TX) to preserve protein integrity. MSCs were lysed using RIPA cell lysis buffer (GenDEPOT). Proteins were quantified using a DC protein quantification kit (Bio‐Rad, Hercules, CA) and protein samples loaded into precast gels (Bio‐Rad). Proteins were separated by SDS‐PAGE at 75 V for 150 min. Gels were then transferred to a nitrocellulose membrane (Bio‐Rad) in an ice bath at 350 mA for 120 min. The membrane was blocked with 5% BSA resuspended in 1X Tris‐buffered saline with 0.1% Tween‐20 (TBST). Subsequently, the membrane was incubated with primary antibodies for NRF1 (Abcam, Boston, MA, 1:1000), TFAM (Cell signaling, Danvers, MA, 1:1000), COXIV (Cell signaling, 1:5000), p53 (SantaCruz, Dallas, TX, 1:1000), p21 (Abcam, 1:500), p16 (Abcam, 1:500), OPA1 (Abcam, 1:5000), Drp1 (Cell Signaling, 1:5000), Mfn2 (Cell Signaling, 1:5000), Fis1 (Santacruz, 1:2000), SOD1 (Cell signaling, 1:1000), TXN1 (Cell signaling, 1:1000), GSR (SantaCruz, 1:500), HKII (Cell signaling, 1:1000), PFKFB3 (Cell signaling, 1:1000), MCT4 (SantaCruz, 1:100), and β‐actin (Cell signaling, 1:10,000) overnight at 4°C. The membrane was then washed three times with TBST for 10 min and incubated with HRP‐conjugated secondary antibodies HRP‐mouse IgG (Cell signaling, 1:10,000) and HRP‐rabbit (Cell signaling, 1:10,000) for 2 h at RT. The membrane was then washed three times for 10 min with TBST and developed with Immobilon HRP substrate (MilliporeSigma). Bands were visualized using the ChemiDoc‐XRS imaging system (Bio‐Rad).

### 4.10 Mitochondrial DNA copy number

MSCs were cultured in 6‐well plates and organized into respective experimental groups. Following mRNA (SCR or NRF1) transfection, MSCs were detached using trypsin, washed twice with PBS, and genomic DNA subsequently isolated utilizing the SpeeDNA Isolation Kit (Sciencell, Carlsbad, CA). Isolated DNA was diluted 1:50 in molecular biology grade water. Quantification of relative mtDNA copy number was performed via real‐time PCR, employing the Human Mitochondrial DNA Copy Number Quantification qPCR Assay Kit (Sciencell) according to the manufacturer's guidelines. In brief, 1 ng of either extracted or reference DNA was mixed with 2 μL of primer (mtDNA or single‐copy reference), 2X GoldNStar TaqGreen qPCR master mix, and 7 μL of nuclease‐free water. The PCR cycling parameters were followed as per the manufacturer's protocol, and the resulting quantification cycle (Cq) values were analyzed using the ^ΔΔ^Cq method. Genomic DNA from a reference sample was used for comparative analysis of relative mtDNA copy number.

### 4.11 Citrate synthase assay

MSCs were seeded in 6‐well plates at a density of 0.8 × 10^4^ cells/cm^2^. The following day, cells underwent either mRNA (SCR or NRF1) transfection, or were treated with bezafibrate (50 μM, 24 h) or doxycycline (10 μg/mL, 24 h), according to the respective experimental protocols. The citrate synthase activity was determined by manufacturer's protocol (Sciencell). Briefly, fresh mitochondria were isolated as described by Wu et al. (2018). Isolated mitochondria were resuspended in Buffer E and distributed into 96‐well plates. After addition of substrate, absorbance was measured at 1 min intervals with a FLUOstar Omega microplate reader (BMG Labtech, Berlin, DE) during 10 min at 412 nm, based on the reaction between 5,5′‐dithiobis‐(2‐nitrobenzoic acid) (DTNB) and CoA‐SH, which forms 2‐nitro‐5‐thiobenzoic acid (TNB).

### 4.12 Mitochondrial ROS, total ROS, and membrane potential measurement

Mitochondrial superoxide accumulation, intracellular total ROS, and membrane potential were visualized using confocal microscopy, while mean fluorescence intensity (MFI) and median fluorescence intensity (MedFI) quantification was determined via flow cytometry analysis using an LSRII Flow Cytometer. Following mRNA transfection, MSCs on collagen‐coated coverslips were stained with red MitoSOX (ThermoFisher Scientific), H2DCFDA (Invitrogen), or JC‐1 (Biotium, Fremont, CA) solution for 15 min and fixed with 4% PFA. Following PBS washing for fluorescence imaging, cells were counter‐stained with DAPI for 15 min and visualized using a Nikon A1 Confocal Imaging System (Nikon). MitoSOX‐stained cells and H2DCFDA‐stained cells were analyzed for TRITC and FITC fluorescence, respectively, and MFI was determined by flow cytometry. JC‐1‐stained cells were examined by flow cytometry for TRITC (J aggregates)‐ and FITC (JC‐1 monomers)‐associated fluorescence. The ratio of J aggregates to JC‐1 monomers was then evaluated as the following: TRITC MedFI/FITC MedFI.

### 4.13 Bioenergetic measurements

MSCs were seeded onto Seahorse XFe96 cell culture microplates (Agilent Technologies, Santa Clara, CA) and after mRNA transfection, a Seahorse XFe96 Analyzer (Agilent) was employed to measure oxygen consumption rate (OCR) and extracellular acidification rate (ECAR), adhering to the manufacturer's guidelines for the Seahorse XF Cell Mito Stress Test Kit (Agilent). OCR and ECAR measurements were normalized using DAPI nuclei staining. Quantification was conducted blindly using ImageJ software version 1.53. The entire well area was documented using a Keyence BZ‐X810 microscope (Keyence, Itasca, IL).

Intracellular ATP levels in MSCs were evaluated utilizing the ATPlite luminescence assay kit (PerkinElmer, Waltham, MA) in accordance with the manufacturer's guidelines. After mRNA transfection, lysis buffer was applied to the cells and agitated at 700 rpm for 5 min. Subsequently, lyophilized substrate solution was mixed at 700 rpm for 5 min. Following a 10‐min incubation in the dark at RT, ATP production was quantified using a luminescence microplate reader (BioTek, Winooski, VT).

l‐lactate levels in the MSC cell culture medium were measured using the l‐lactate assay kit (Abcam). Briefly, 200 μL of cell culture supernatant was collected at the end of the experiment and diluted 40‐fold with assay buffer. Optical density was then measured at 570 nm with a FLUOstar Omega microplate reader. The l‐lactate concentration was determined by comparing the optical density to a standard curve, which was generated using known concentrations of l‐lactate standards, following the manufacturer's protocol.

### 4.14 Mitochondrial network analysis

Mitochondrial fragmentation was evaluated in mitoAcGFP1‐expressing MSCs or Mitotracker^TM^ Deep Red FM (Invitrogen)‐stained MSCs using confocal microscopy fluorescence imaging. Quantification of mitochondrial fragmentation was performed using the Mitochondrial Fragmentation Count (MFC) analysis tool in ImageJ. This consisted of converting confocal images of single cells to binary images, followed by counting of discrete and non‐contiguous particles (i.e., mitochondria). MFC was calculated by dividing the number of particles by the number of black pixels, and arbitrarily multiplying by 1000, following a methodology outlined in a prior report (Valente et al., 2017).

### 4.15 SA‐β‐gal activity

To assess SA‐β‐gal activity after mRNA transfection, MSCs were washed with PBS and fixed with a fixation solution composed of 2% formaldehyde (vol/vol) and 0.2% glutaraldehyde (vol/vol) in PBS for 5 min. Following fixation, cells underwent an 8‐h incubation at 37°C in a CO_2_‐free environment, and then immersed in a prepared X‐gal staining solution consisting of X‐gal (Invitrogen, Waltham, MA), citric acid/Na phosphate buffer (40 mM pH 6.0), potassium hexacyanoferrate (II) trihydrate (5 mM), potassium hexacyanoferrate (III) (5 mM), NaCl (150 mM), and MgCl_2_ (2 mM). After staining, cells were washed with PBS and images taken using the EVOS cell imaging system. SA‐β‐gal‐positive cells were manually counted and expressed as a percent of total cell number.

### 4.16 Proliferation assay

To assess MSC proliferation, 6 × 10^3^ cells were seeded onto a 96‐well plate. This density was determined necessary to avoid contact inhibition, ensuring accurate evaluation of proliferation over 7 days. After mRNA transfection, MSCs were gently washed twice with PBS and stained with 1 μM of DAPI for 15 min. DAPI‐positive cells were captured by EVOS microscopy and DAPI positive cell number was quantified using Image J software, marking day 0 of the assessment. Subsequent counts and analysis were performed on Days 1, 3, 5, and 7. The number of cell population doubling (NCPD) was calculated based on a previously published method (Yang, Ogando, et al., 2018).

### 4.17 Statistical analysis

The data are presented as mean ± standard error of the mean (SEM). Statistical analysis was conducted using GraphPad Prism 9.5.1 (GraphPad Software, San Diego, CA). Unless otherwise stated in the figure legend, the unpaired one‐way ANOVA analysis method followed by Dunnett's multiple comparison tests was used for all statistical analysis. Statistical significance was denoted as *p < 0.05, **p < 0.005, ***p < 0.0005, and ****p < 0.0001.

## AUTHOR CONTRIBUTIONS

Hyunho Lee designed and performed experiments, analyzed data, constructed schematics, and wrote the manuscript. Matteo Massaro designed and performed experiments, analyzed data, constructed schematics, and revised the manuscript. Gherardo Baudo designed and performed experiments, analyzed data, and revised the manuscript. Nourhan Abdelfattah and Haoran Liu analyzed data and revised the manuscript. Kyuson Yun revised the manuscript. Elvin Blanco designed experiments, analyzed data, and wrote the manuscript.

## FUNDING INFORMATION

This work was supported by the Houston Methodist Research Institute.

## CONFLICT OF INTEREST STATEMENT

None declared.

## Supporting information


**Data S1.**.

## Data Availability

The data that support the findings of this study are available from the corresponding author upon reasonable request.
