# Supplementary material for: Nuclear respiratory factor‐1 (NRF1) induction as a powerful strategy to deter mitochondrial dysfunction and senescence in mesenchymal stem cells
Source: Aging Cell. 2024 Dec 25;24(4):e14446. doi: 10.1111/acel.14446 (PMC11984659; doi:10.1111/acel.14446)
Supplement: Supplementary file 1 — Data S1.. [file ACEL-24-e14446-s001.docx]

**Supplementary Materials**

**Nuclear respiratory factor-1 (NRF1) induction as a powerful strategy to deter mitochondrial dysfunction and senescence in mesenchymal stem cells**

Hyunho Lee, Matteo Massaro, Nourhan Abdelfattah, Gherardo Baudo, Haoran Liu,

Kyuson Yun, and Elvin Blanco*


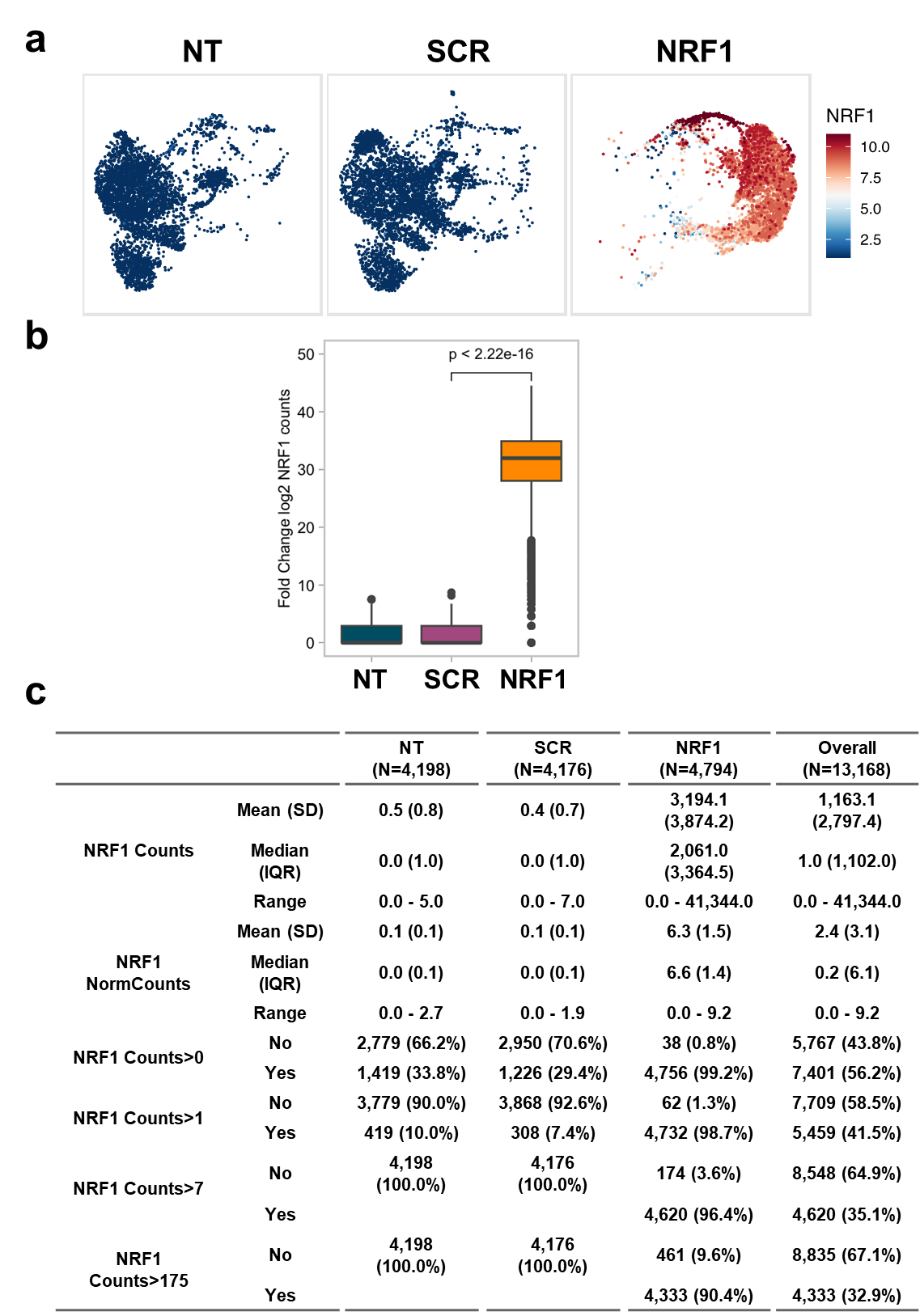


**Figure S1.** **NRF1 overexpression in MSCs resulted in a high transfection efficiency of NRF1.** MSCs were transfected with either scrambled (SCR) or NRF1 mRNA. Controls consisted of non-transfected MSCs (NT). a) Feature plot showing normalized expression of NRF1 across NT, SCR, and NRF1 mRNA-transfected MSCs. b) Box plot demonstrating fold change of Log2 NRF1 counts among the different groups relative to the mean log2 NRF1 expression in SCR cells. c) Tabulated summary of NRF1 counts among the different groups at the single-cell level, with NRF1 expression at different count thresholds.


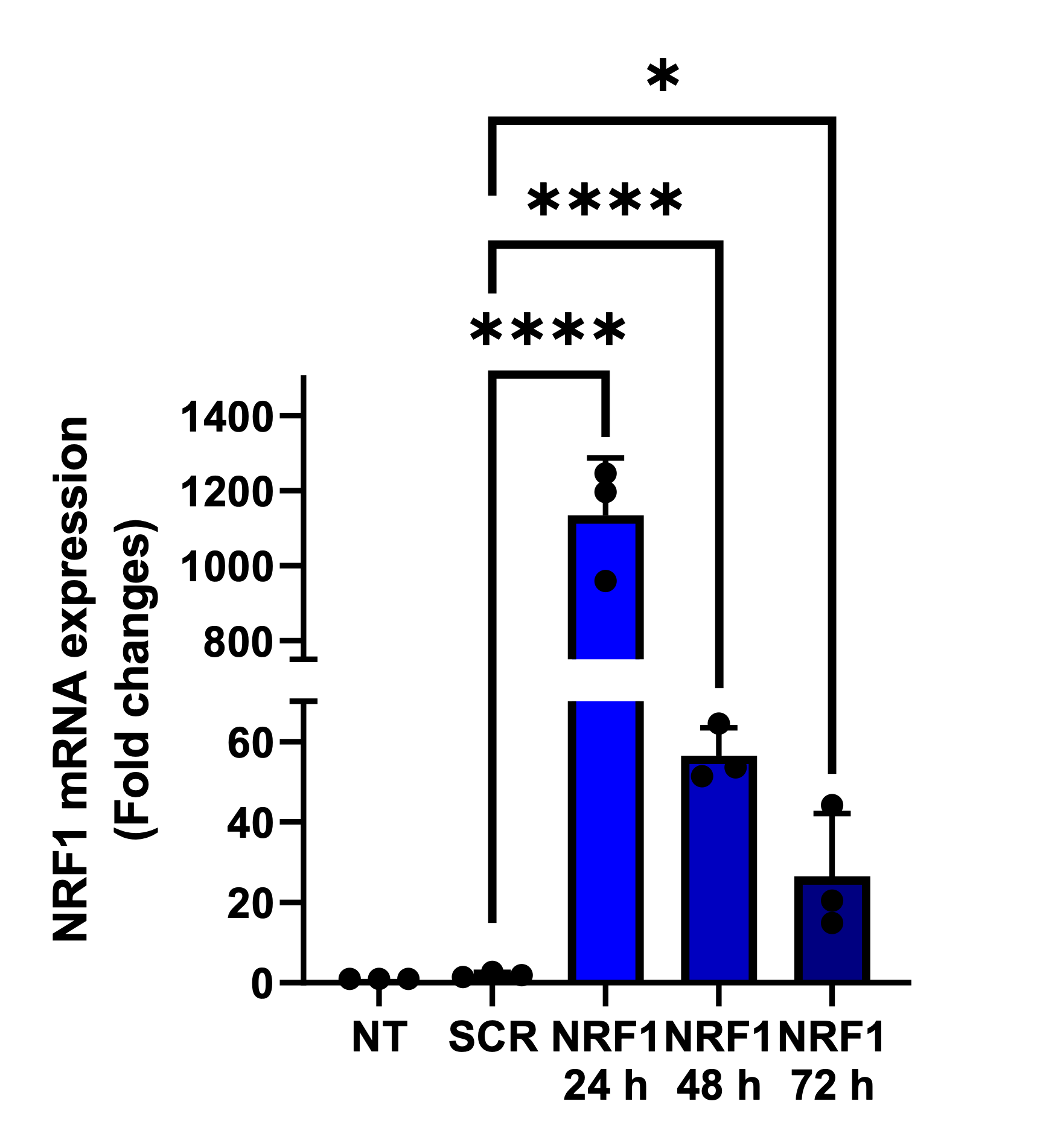


**Figure S2. Time course analysis of NRF1 mRNA expression in MSCs as determined by RT-qPCR.**MSCs were transfected with either scrambled (SCR) or NRF1 mRNA and total RNA harvested at different timepoints. Controls consisted of non-transfected MSCs (NT). Significance was determined using a one-tailed unpaired t-test between SCR and NRF1 groups. *p<0.05; ****p<0.0001.


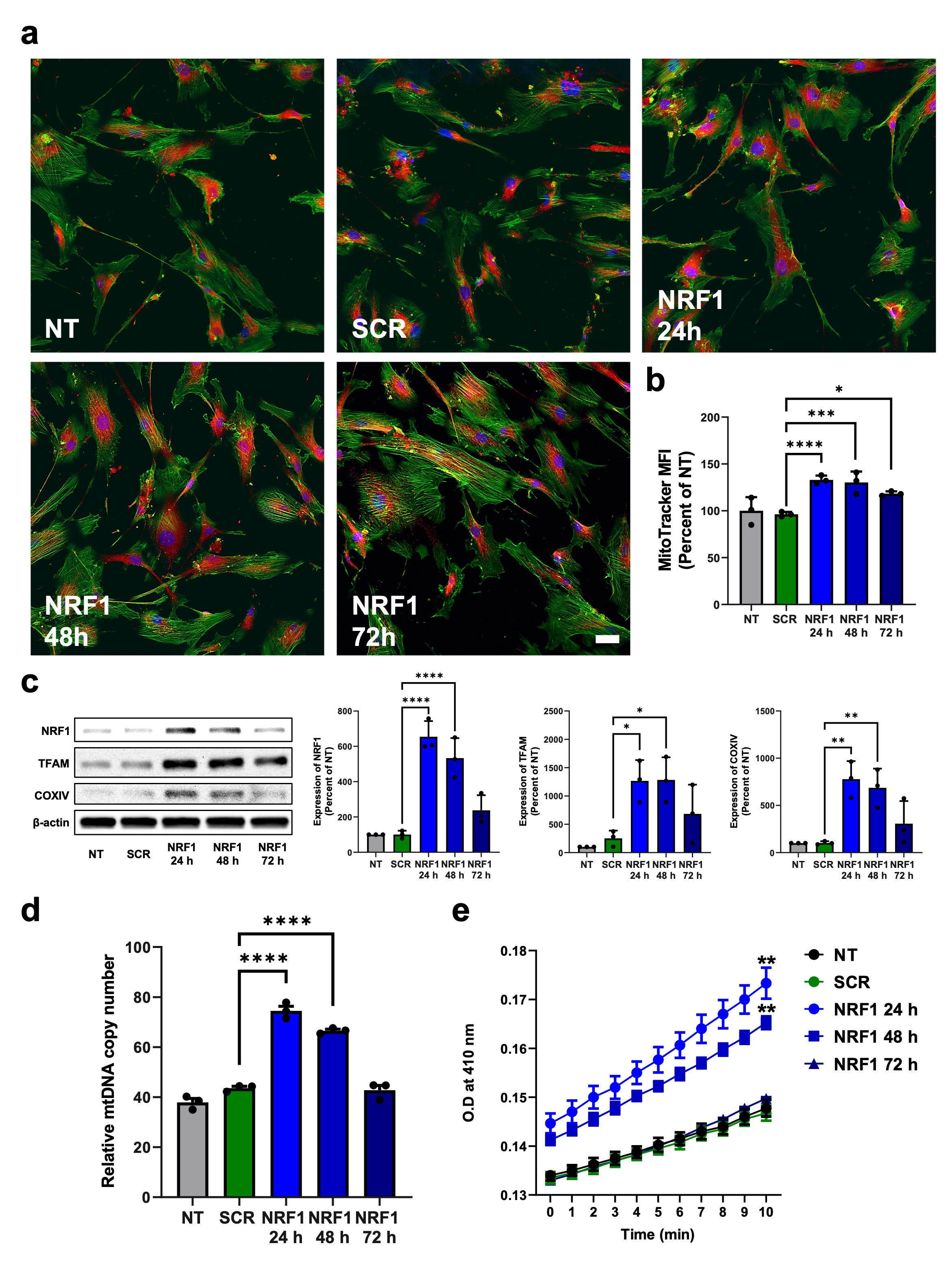


**Figure S3. Time course analysis of NRF1-induced mitochondrial biogenesis.** MSCs were transfected with either scrambled (SCR) or NRF1 mRNA and cells harvested at different timepoints. Controls consisted of non-transfected MSCs (NT). a) Representative confocal microscopy images depicting MitoTracker-stained mitochondria in MSCs. Red represents MitoTracker-associated fluorescence, green represents F-actin staining, and blue represents DAPI nuclear staining. Scale bar = 50 μm. b) MitoTracker mean fluorescence intensity (MFI) quantified by flow cytometry analysis. c) Representative western blot of NRF1, TFAM, and COXIV expression in MSCs. Densitometric analysis was performed to quantify protein expression. Protein markers were normalized to β-actin expression levels. d) Quantification of relative mitochondrial DNA copy number (mtDNA) by qRT-PCR. e) Citrate synthase activity. *p<0.05; **p<0.005; ***p<0.0005; ****p<0.0001. For citrate synthase activity, two-way ANOVA followed by Tukey’s multiple comparison test was used for statistical analysis: **p<0.005 vs SCR group.


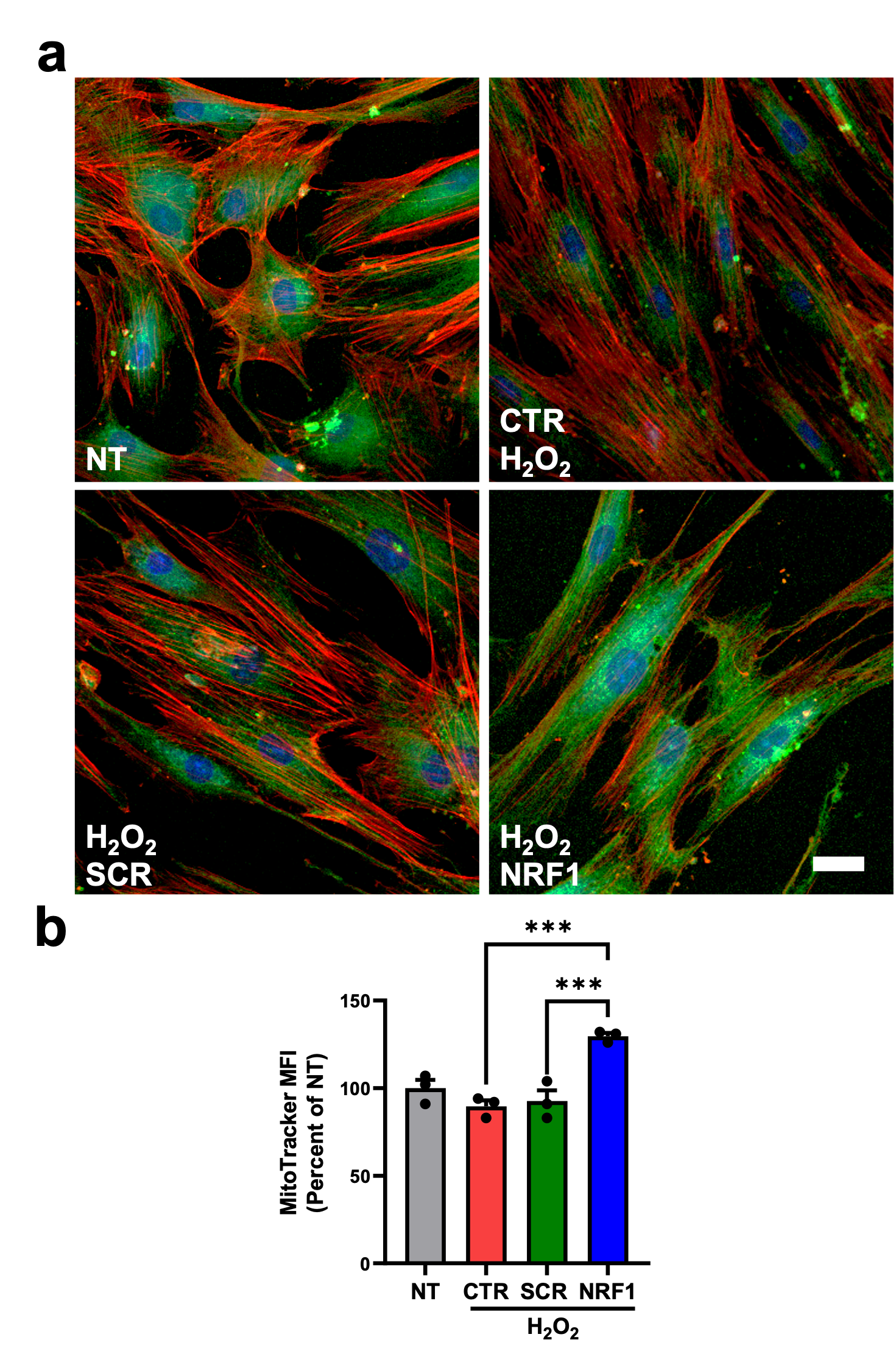


**Figure S4. NRF1 overexpression increased mitochondrial mass in MSCs exposed to oxidative stress.** H_2_O_2_-exposed (250 µM, 1 h) MSCs were transfected with either scrambled (SCR) or NRF1 mRNA. Controls consisted of non-transfected MSCs (NT) and NT MSCs exposed to H_2_O_2_ (CTR). a) Representative confocal microscopy images depicting MitoTracker-stained mitochondria in MSCs. Green represents MitoTracker-associated fluorescence, red represents F-actin staining, and blue represents DAPI nuclear staining. Scale bar = 15 μm. b) MitoTracker mean fluorescence intensity (MFI) quantified by flow cytometry. ***p<0.0005.


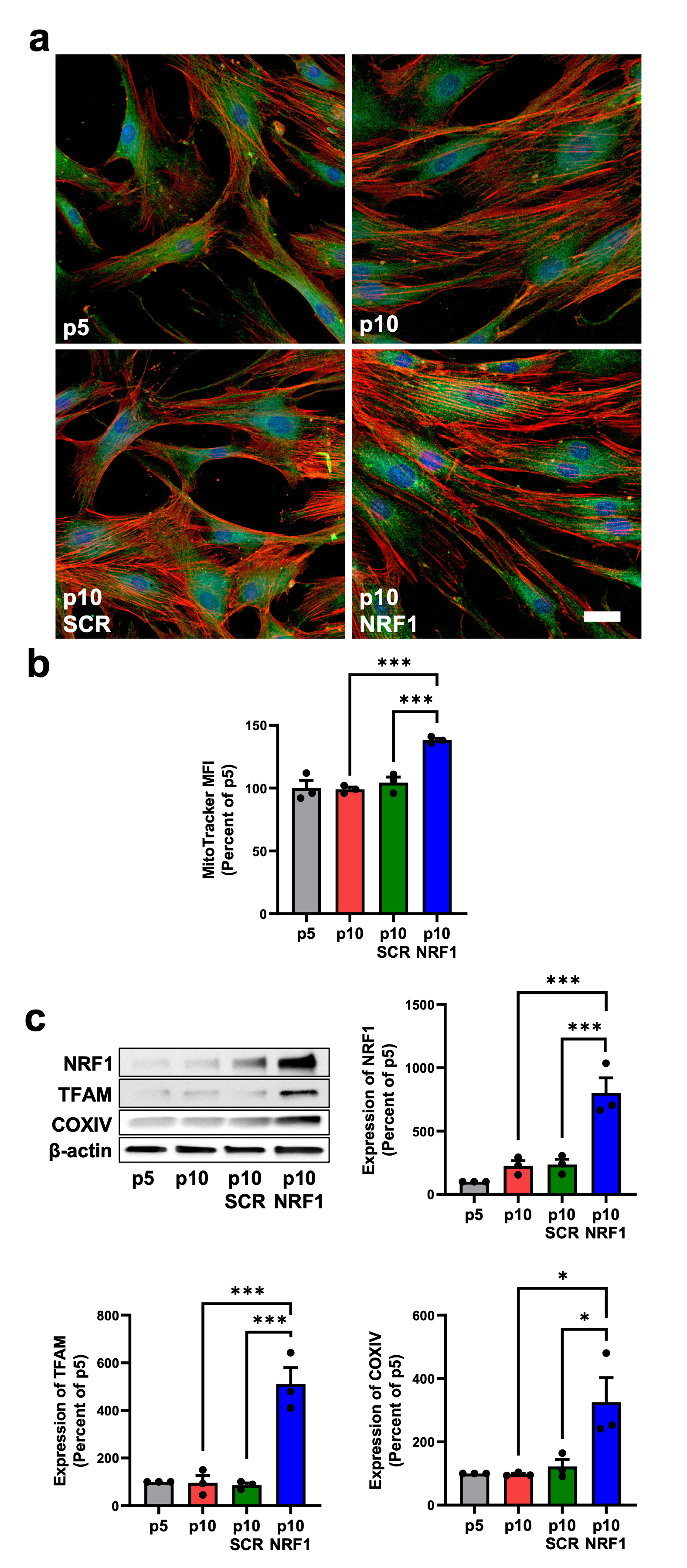


**Figure S5.** **NRF1 overexpression increased mitochondrial mass in MSCs undergoing replicative senescence.** MSCs at passage 10 (p10) were transfected with either scrambled (SCR) or NRF1 mRNA. Controls consisted of non-transfected MSCs at passages 5 (p5) and 10 (p10). a) Representative confocal microscopy images depicting MitoTracker-stained mitochondria in MSCs. Green represents MitoTracker-associated fluorescence, red represents F-actin staining, and blue represents DAPI nuclear staining. Scale bar = 15 μm. b) MitoTracker mean fluorescence intensity (MFI) quantified by flow cytometry. c) Representative western blot of NRF1, TFAM, and COXIV expression in MSCs. Densitometric analysis was performed to quantify protein expression. Protein markers were normalized to β-actin expression levels. *p<0.05, ***p<0.0005.


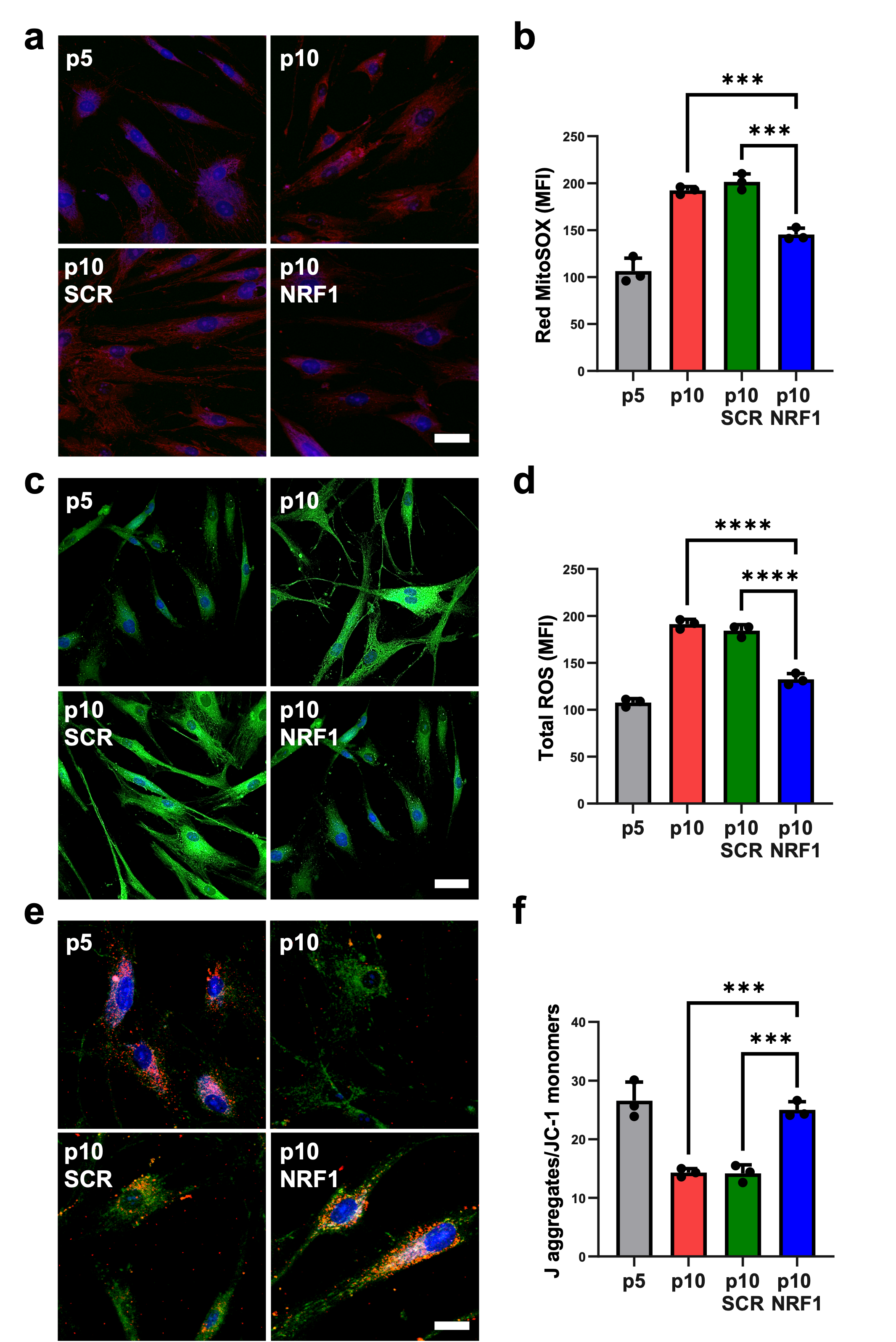


**Figure S6. NRF1 overexpression reduced ROS and preserved mitochondrial health in MSCs undergoing replicative senescence.** MSCs at passage 10 (p10) were transfected with either scrambled (SCR) or NRF1 mRNA. Controls consisted of non-transfected MSCs at passages 5 (p5) and 10 (p10). a) Representative confocal microscopy images depicting MitoSOX stained MSCs. Oxidized MitoSOX reagent is represented in red and DAPI-stained nuclei appear in blue. Scale bar = 15 µm. (b) Mean fluorescence intensity (MFI) of oxidized MitoSOX reagent quantified by flow cytometry. c) Representative confocal microscopy images depicting intracellular total ROS via H2DCFDA staining. Oxidized H2DCFDA is represented in green and DAPI-stained nuclei appear in blue. Scale bar = 50 µm. d) MFI of oxidized H2DCFDA quantified by flow cytometry. e) Representative confocal microscopy images of MSCs undergoing JC-1 staining. JC-1 monomers are represented in green, J aggregates in red, and DAPI-stained nuclei in blue. Scale bar = 15 µm. f) The ratio of the median fluorescence intensity (MedFI) of J aggregates to JC-1 monomers quantified by flow cytometry. ***p<0.0005; ****p<0.0001.


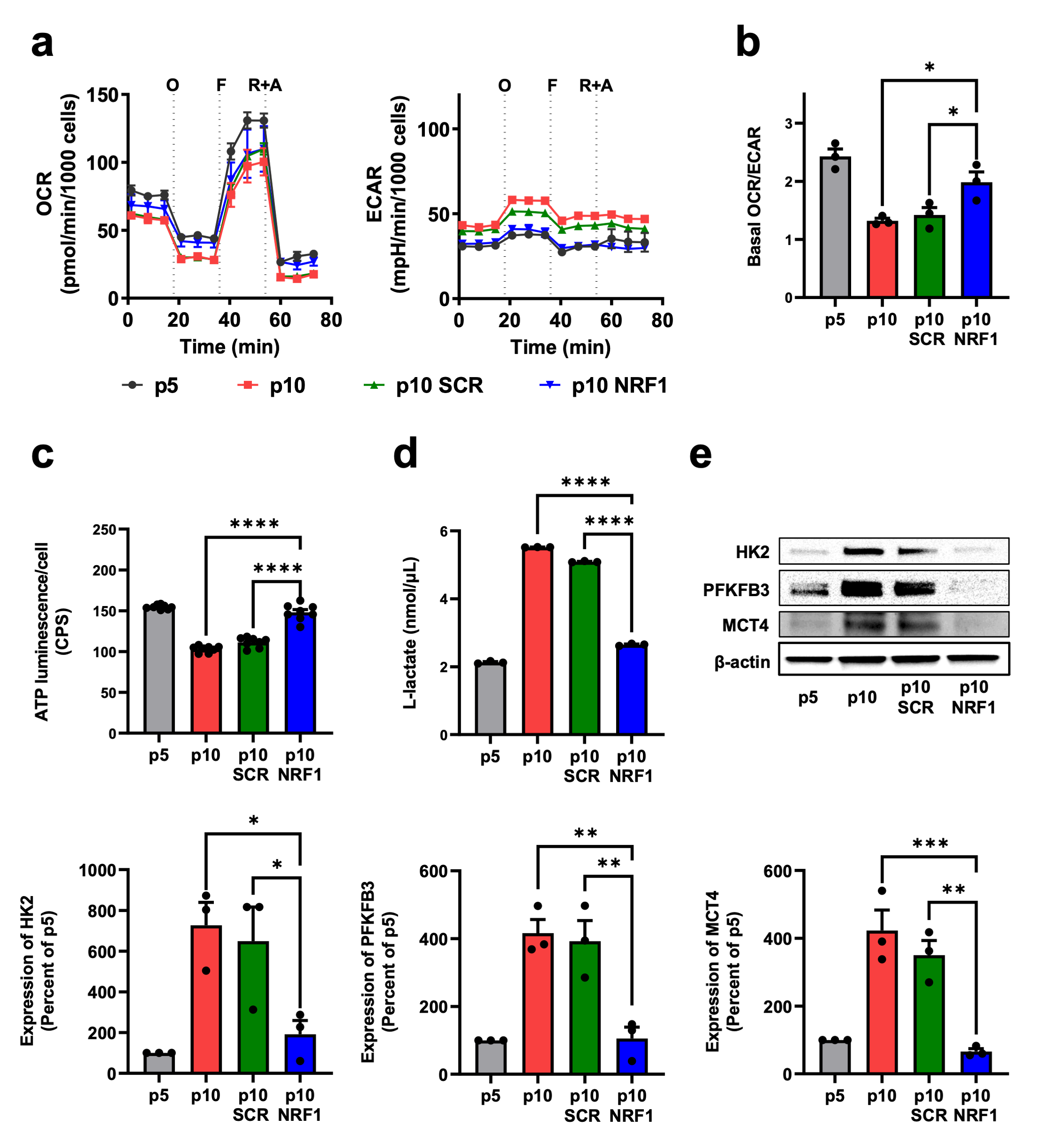


**Figure S7. NRF1 overexpression enhanced cell bioenergetics and reduced glycolysis in MSCs undergoing replicative senescence.** MSCs at passage 10 (p10) were transfected with either scrambled (SCR) or NRF1 mRNA. Controls consisted of non-transfected MSCs at passages 5 (p5) and 10 (p10). a) Bioenergetic analysis of the effect of NRF1 overexpression in MSCs via examination of the oxygen consumption rate (OCR) and extracellular acidification rate (ECAR). O: oligomycin; F: FCCP; R + A: rotenone + antimycin A. b) The ratio of basal OCR/ECAR of MSCs. c) Relative intracellular ATP. d) L-lactate production. e) Representative western blot of HK2, PFKFB3, and MCT4 expression in MSCs. Densitometric analysis was performed to quantify protein expression. Protein markers were normalized to β-actin expression levels. *p<0.05; **p<0.005; ***p<0.0005 ****p<0.0001.


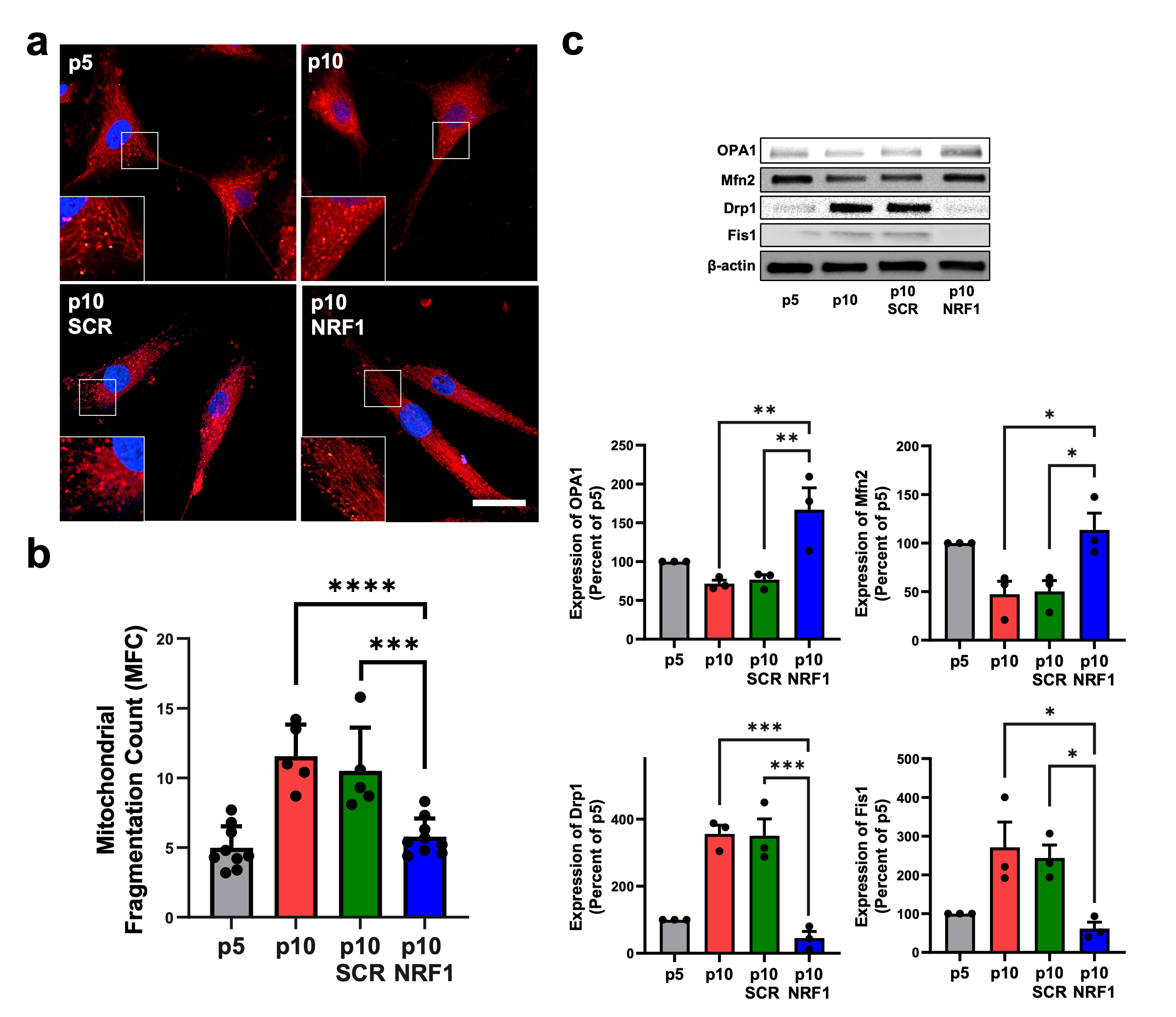


**Figure S8. NRF1 overexpression preserved mitochondrial dynamics in MSCs undergoing replicative senescence.** MSCs at passage 10 (p10) were transfected with either scrambled (SCR) or NRF1 mRNA. Controls consisted of non-transfected MSCs at passages 5 (p5) and 10 (p10). a) Representative confocal microscopy images of MitoTracker-stained mitochondrial morphology in MSCs. Red represents MitoTracker associated fluorescence and blue represents DAPI nuclear staining. Scale bar = 50 µm. b) Mitochondrial fragmentation count (MFC). c) Representative western blot analysis of mitochondrial dynamics-related proteins (OPA1, Mfn2, Drp1, and Fis1) in MSCs. Densitometric analysis was performed to quantify protein expression, normalized to β-actin levels. *p<0.05; **p<0.005; ***p<0.0005; ****p<0.0001.


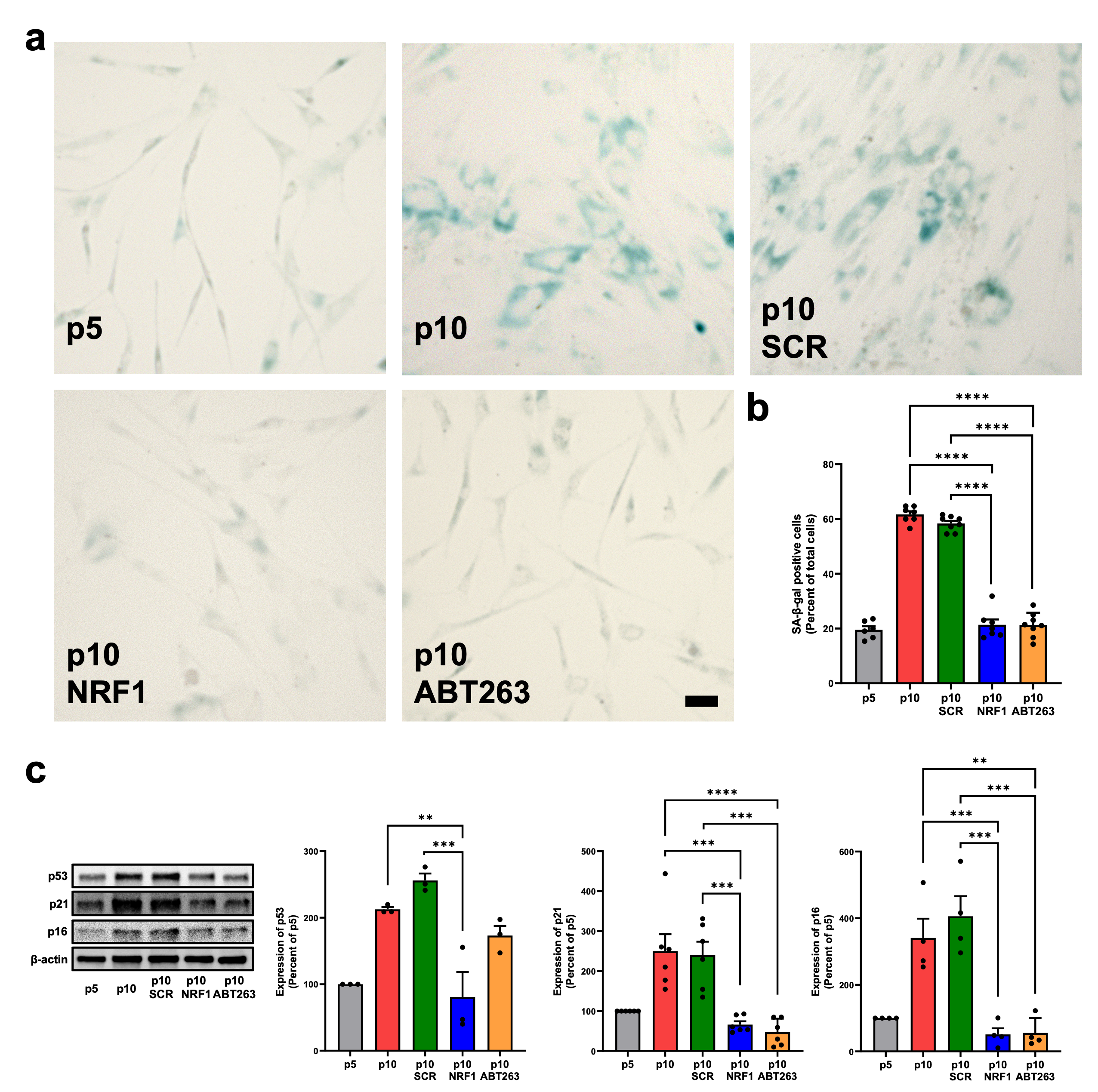


**Figure S9. NRF1 overexpression in MSCs suppressed replicative senescence.** MSCs at passage 10 (p10) were either transfected with scrambled (SCR) or NRF1 mRNA, or treated with ABT263. Controls consisted of non-transfected/non-treated MSCs at passages 5 (p5) and 10 (p10). a) Representative light microscopy images of SA-β-gal activity in MSCs. Scale bar = 50 µm. b) SA-β-gal activity expressed as the percentage of SA-β-gal positive cells. c) Representative western blot of p53, p21, and p16 expression in MSCs. Densitometric analysis was performed to quantify protein expression, with all protein markers normalized to β-actin expression levels. **p<0.005; ***p<0.0005; ****p<0.0001.


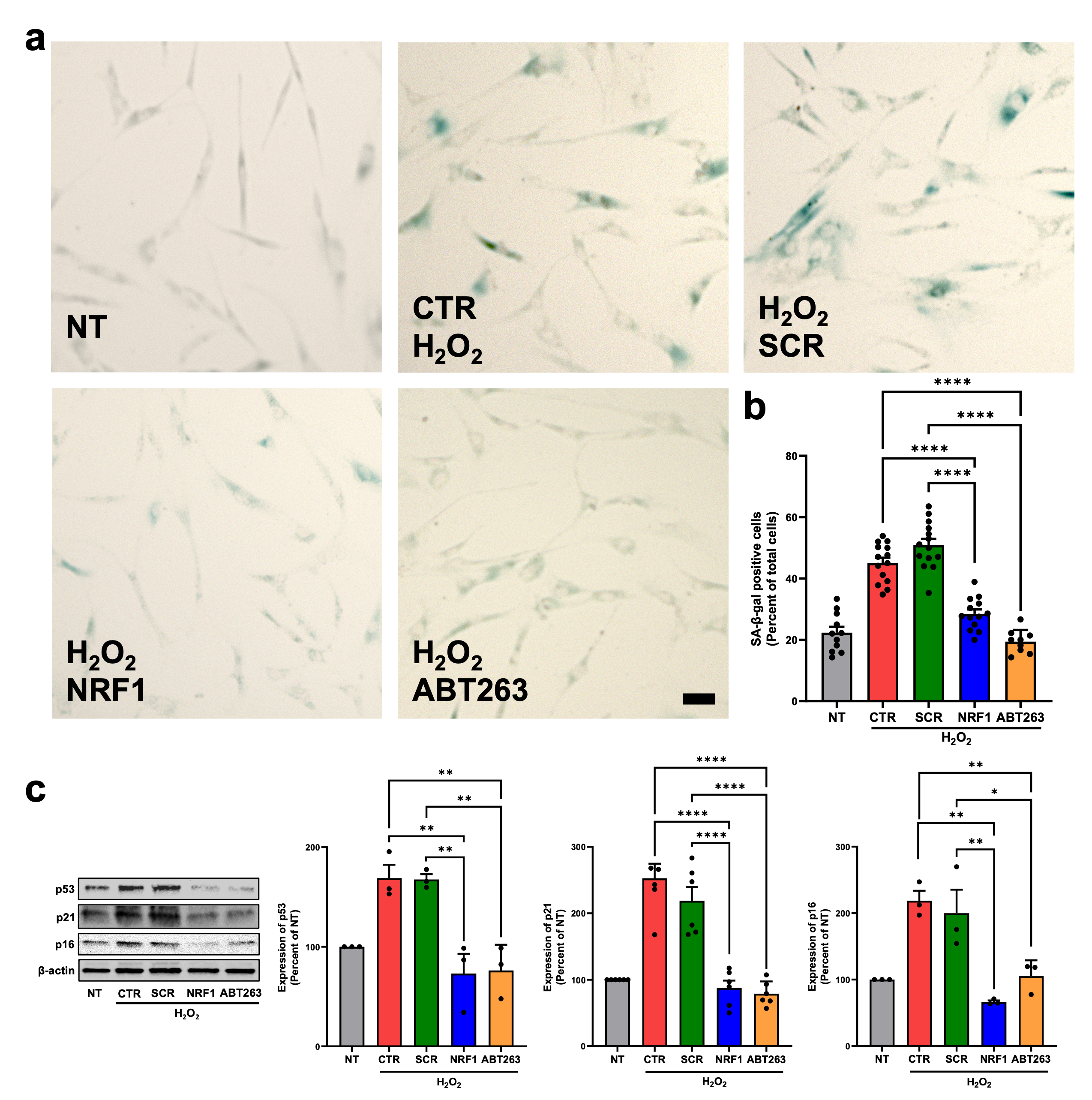


**Figure S10. Effect of NRF1 overexpression on oxidative stress-induced senescence in MSCs compared to ABT263.** H_2_O_2_-exposed (250 µM, 1 h) MSCs were either transfected with scrambled (SCR) or NRF1 mRNA, or treated with ABT263. Controls consisted of non-transfected/non-treated MSCs (NT) and NT MSCs exposed to H_2_O_2_ (CTR). a) Representative light microscopy images of SA-β-gal activity in MSCs. Scale bar = 50 µm. b) SA-β-gal activity expressed as the percentage of SA-β-gal positive cells. c) Representative western blot of p53, p21, and p16 expression in MSCs. Densitometric analysis was performed to quantify protein expression, with all protein markers normalized to β-actin expression levels. *p<0.05; **p<0.005; ****p<0.0001.


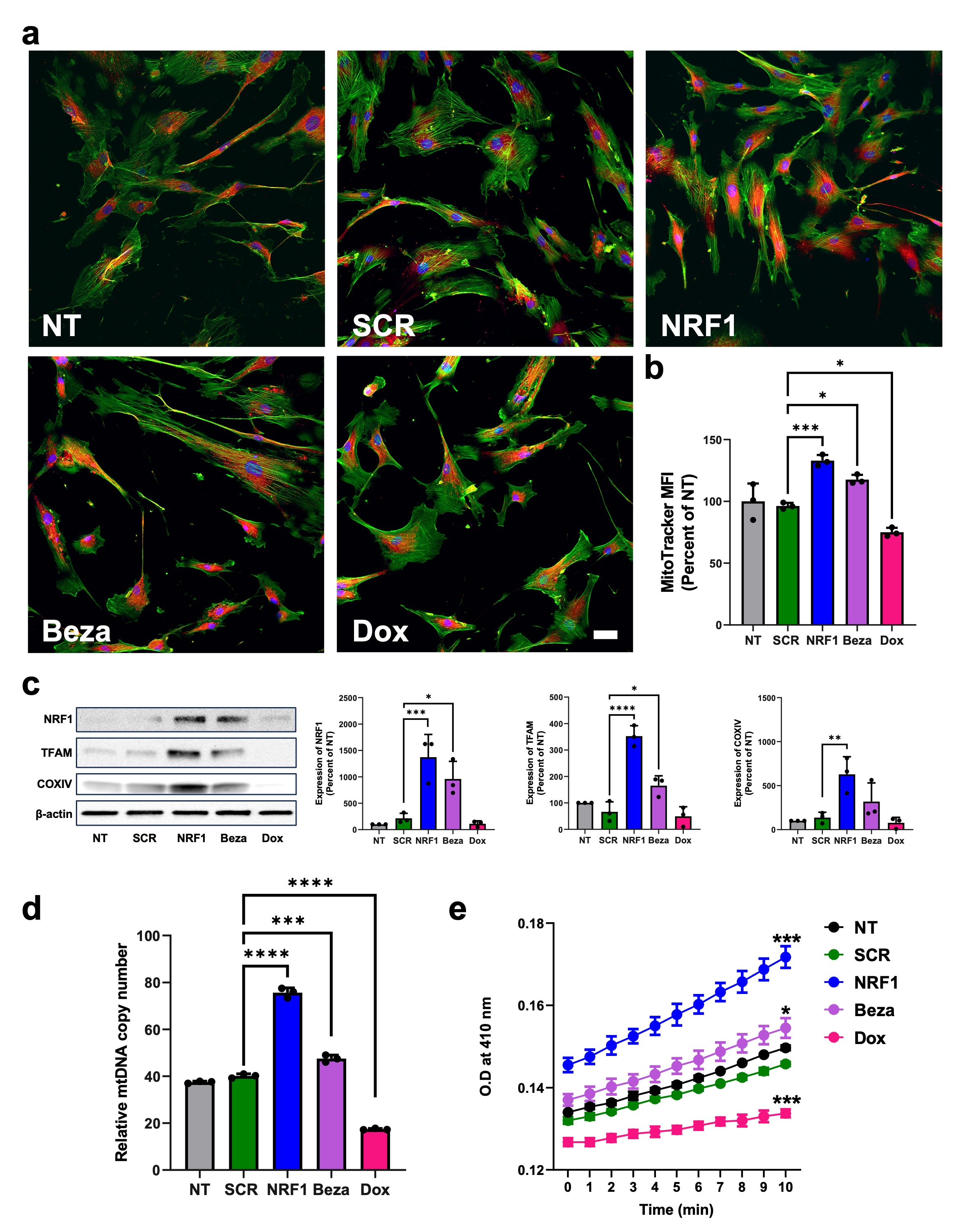


**Figure S11. Effects of bezafibrate and doxycycline on mitochondrial biogenesis.** MSCs were either transfected with scrambled (SCR) or NRF1 mRNA, or treated with bezafibrate or doxycycline. Controls consisted of non-transfected MSCs (NT). a) Representative confocal microscopy images depicting MitoTracker-stained mitochondria in MSCs. Red represents MitoTracker-associated fluorescence, green represents F-actin staining, and blue represents DAPI nuclear staining. Scale bar = 50 μm. b) MitoTracker mean fluorescence intensity (MFI) quantified by flow cytometry analysis. c) Representative western blot of NRF1, TFAM, and COXIV expression in MSCs. Densitometric analysis was performed to quantify protein expression. Protein markers were normalized to β-actin expression levels. d) Quantification of relative mitochondrial DNA copy number (mtDNA) by qRT-PCR. e) Citrate synthase activity. *p<0.05; **p<0.005; ***p<0.0005; ****p<0.0001. For citrate synthase activity, two-way ANOVA followed by Tukey’s multiple comparison test was used for statistical analysis: *p<0.05; ***p<0.0005 vs SCR group.


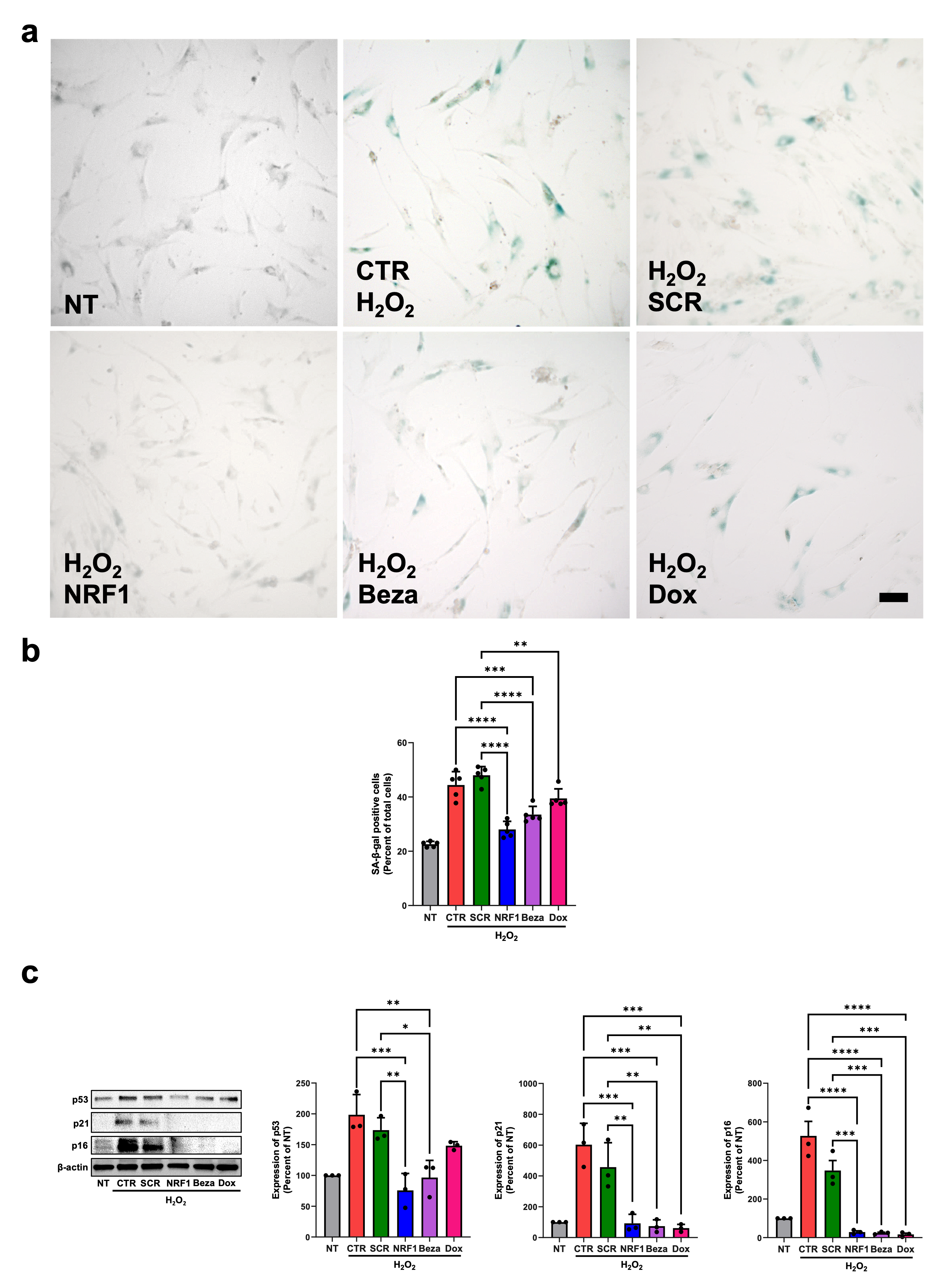


**Figure S12. Bezafibrate and doxycycline effects on stress-induced premature senescence compared to NRF1 overexpression.** H_2_O_2_-exposed (250 µM, 1 h) MSCs were either transfected with scrambled (SCR) or NRF1 mRNA, or treated with bezafibrate or doxycycline. Controls consisted of non-transfected/non-treated MSCs (NT) and NT MSCs exposed to H_2_O_2_ (CTR). a) Representative light microscopy images of SA-β-gal activity in MSCs. Scale bar = 50 µm. b) SA-β-gal activity expressed as the percentage of SA-β-gal positive cells. c) Representative western blot of p53, p21, and p16 expression in MSCs. Densitometric analysis was performed to quantify protein expression, with all protein markers normalized to β-actin expression levels. *p<0.05; **p<0.005; ***p<0.0005; ****p<0.0001.


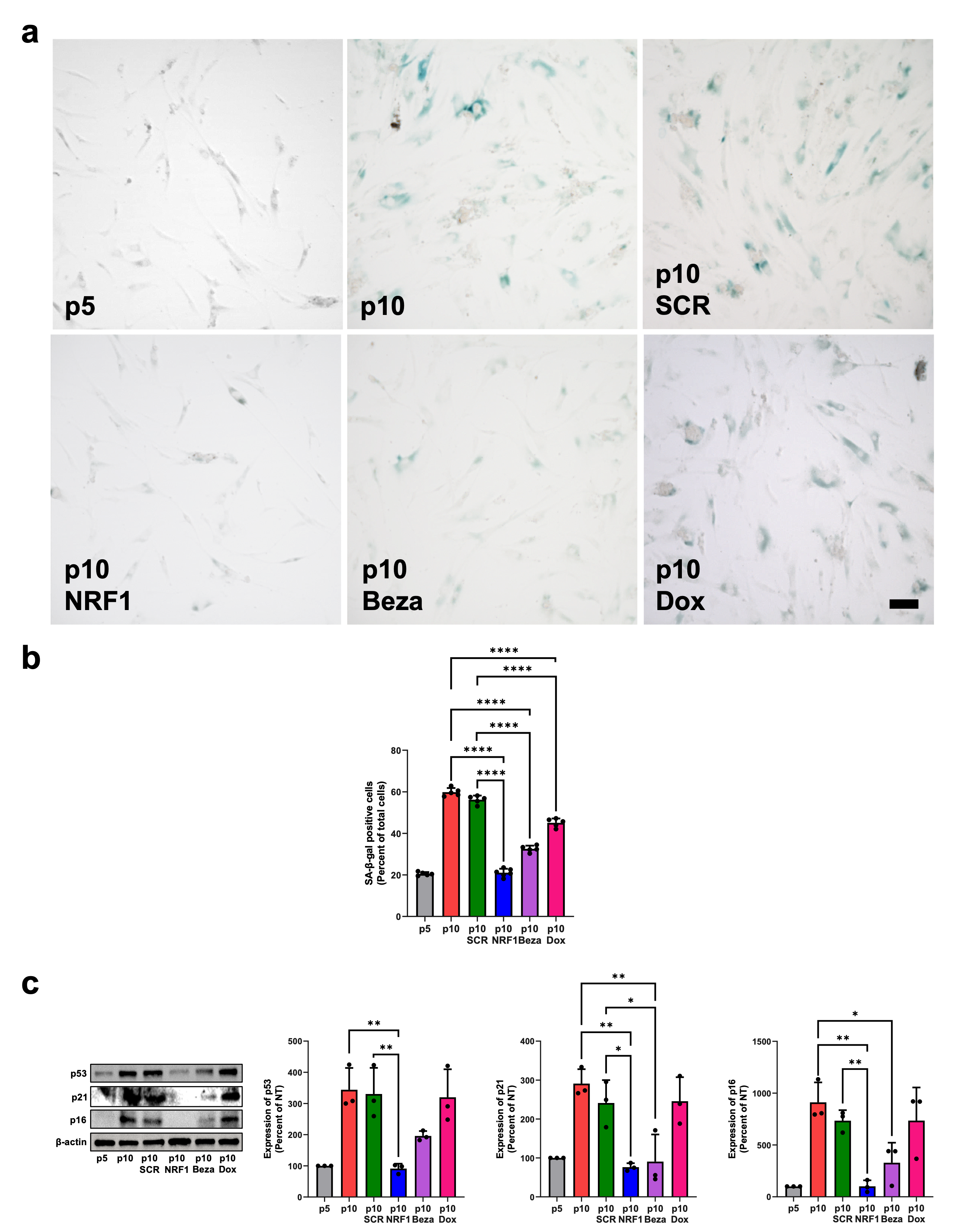


**Figure S13. Bezafibrate and doxycycline effects on replicative senescence compared to NRF1 overexpression.** MSCs at passage 10 (p10) were either transfected with scrambled (SCR) or NRF1 mRNA, or treated with bezafibrate or doxycycline. Controls consisted of non-transfected/non-treated MSCs at passages 5 (p5) and 10 (p10). a) Representative light microscopy images of SA-β-gal activity in MSCs. Scale bar = 50 µm. b) SA-β-gal activity expressed as the percentage of SA-β-gal positive cells. c) Representative western blot of p53, p21, and p16 expression in MSCs. Densitometric analysis was performed to quantify protein expression, with all protein markers normalized to β-actin expression levels. *p<0.05; **p<0.005; ****p<0.0001.


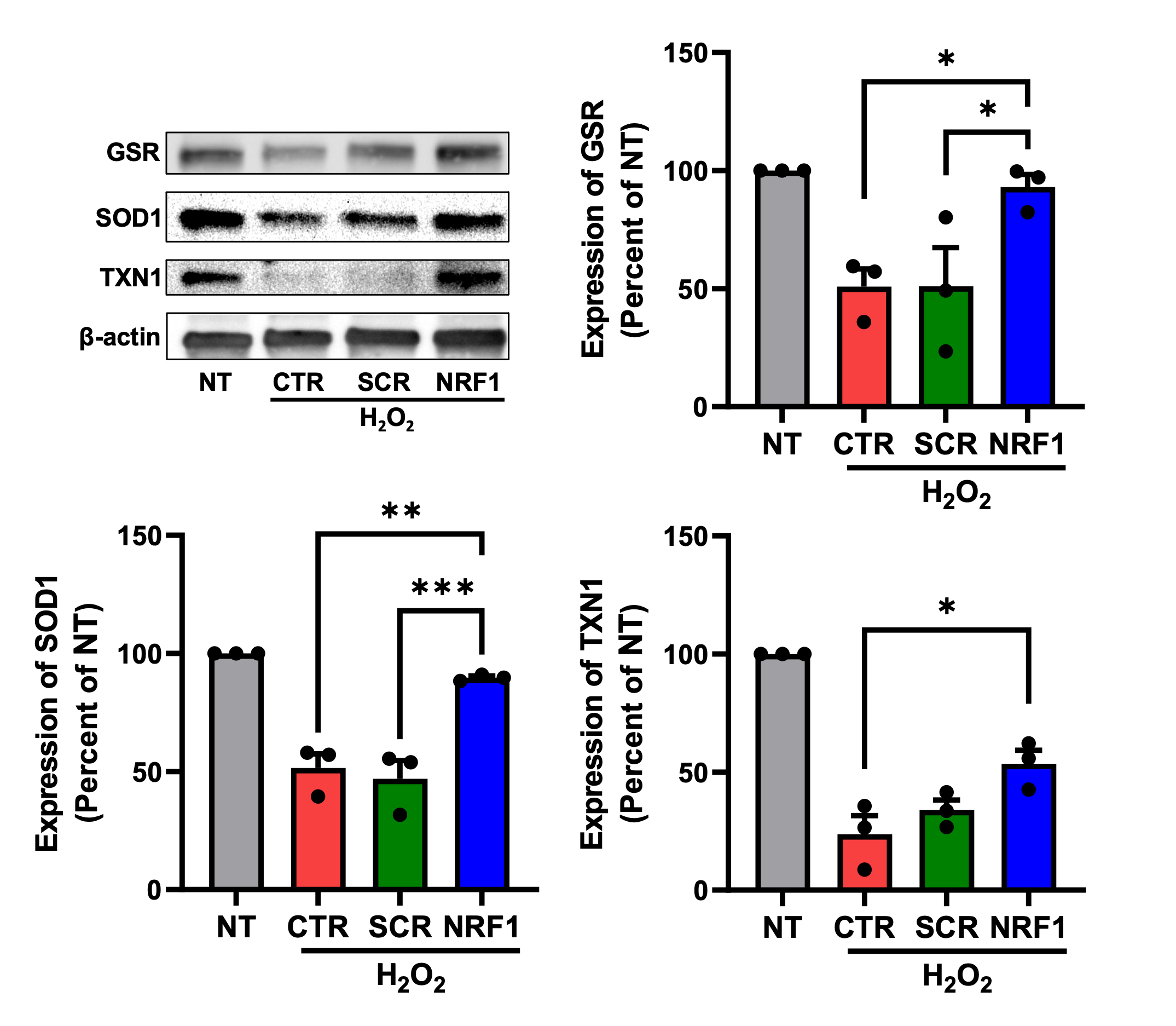


**Figure S14.** **NRF1 overexpression increased the expression of antioxidant enzymes in MSCs exposed to oxidative stress.** H_2_O_2_-exposed (250 µM, 1 h) MSCs were transfected with either scrambled (SCR) or NRF1 mRNA. Controls consisted of non-transfected MSCs (NT) and NT MSCs exposed to H_2_O_2_ (CTR). Representative western blot showing the expression of antioxidant proteins GSR, SOD1, and TXN1 in MSCs. Densitometric analysis was performed to quantify the expression of these antioxidant proteins, normalized to β-actin levels. *p<0.05; **p<0.005; ***p<0.0005.


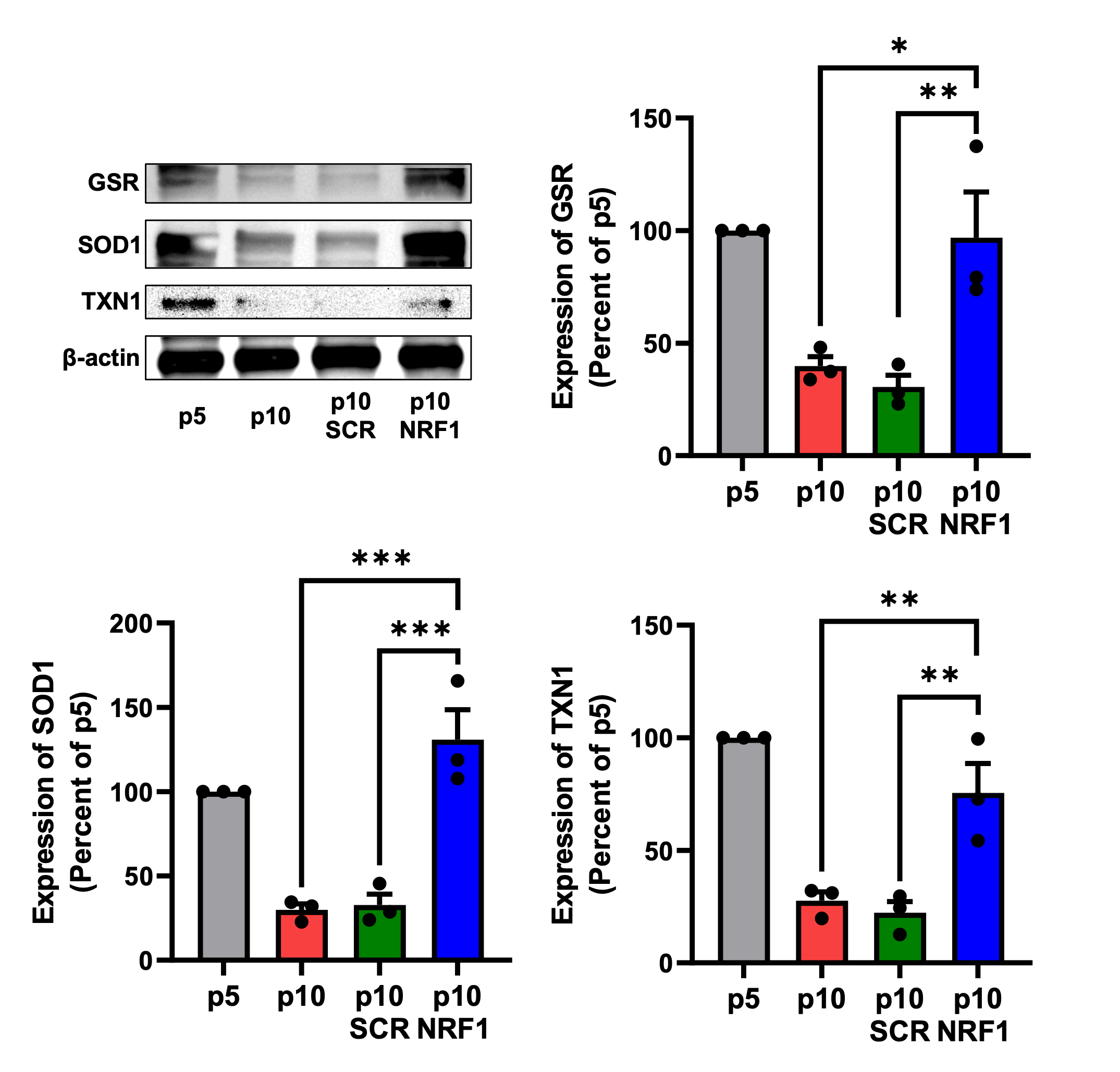


**Figure S15.** **NRF1 overexpression increased the expression of antioxidant enzymes in MSCs undergoing replicative senescence.** MSCs at passage 10 (p10) were transfected with either scrambled (SCR) or NRF1 mRNA. Controls consisted of non-transfected MSCs at passages 5 (p5) and 10 (p10). Representative western blot showing the expression of antioxidant proteins GSR, SOD1, and TXN1 in MSCs. Densitometric analysis was performed to quantify the expression of these antioxidant proteins, normalized to β-actin levels. *p<0.05; **p<0.005; ***p<0.0005.


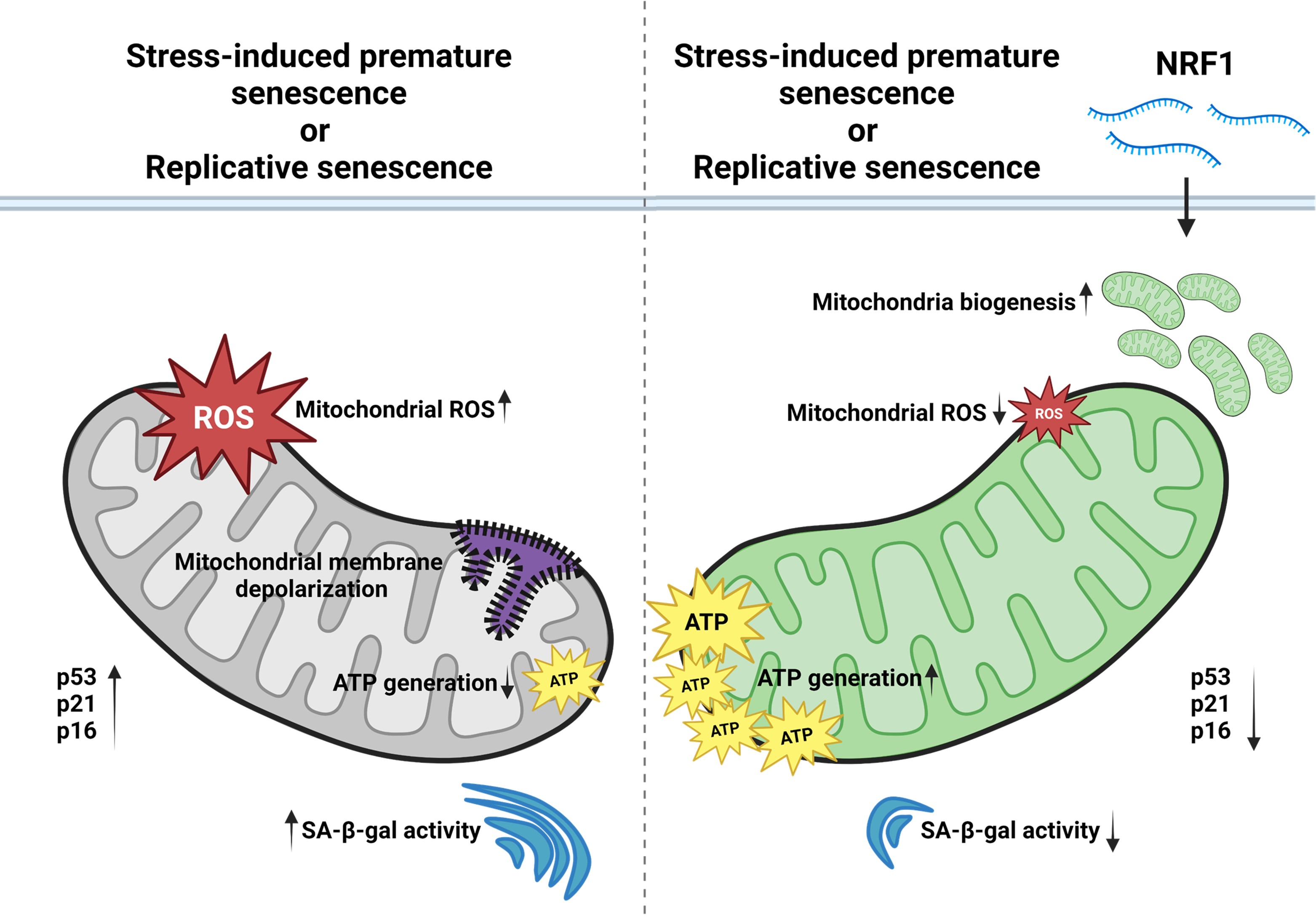


**Figure S16.** **Schematic summary of senescence attenuation in MSCs following NRF1 overexpression**. Stress-induced premature senescence and replicative senescence is associated with mitochondrial dysfunction. NRF1 priming of MSCs reduced ROS and preserved mitochondrial function in MSCs, staving off senescence processes.


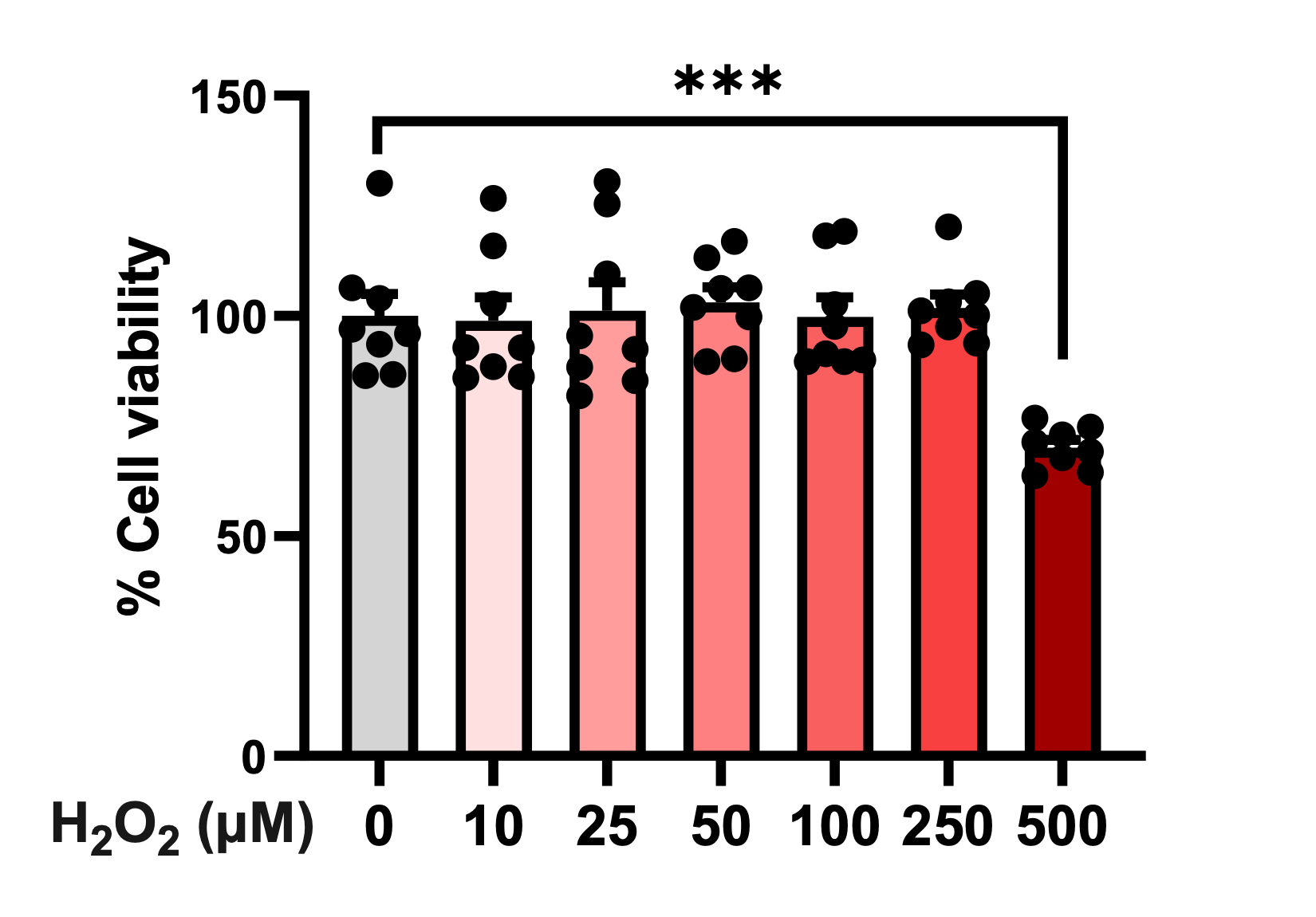


**Figure S17. Effect of H_2_O_2_ concentration on MSC viability.** MSC viability following a 1 h exposure to different concentrations of H_2_O_2_ was determined via MTT assay after 24h. ***p<0.0005.


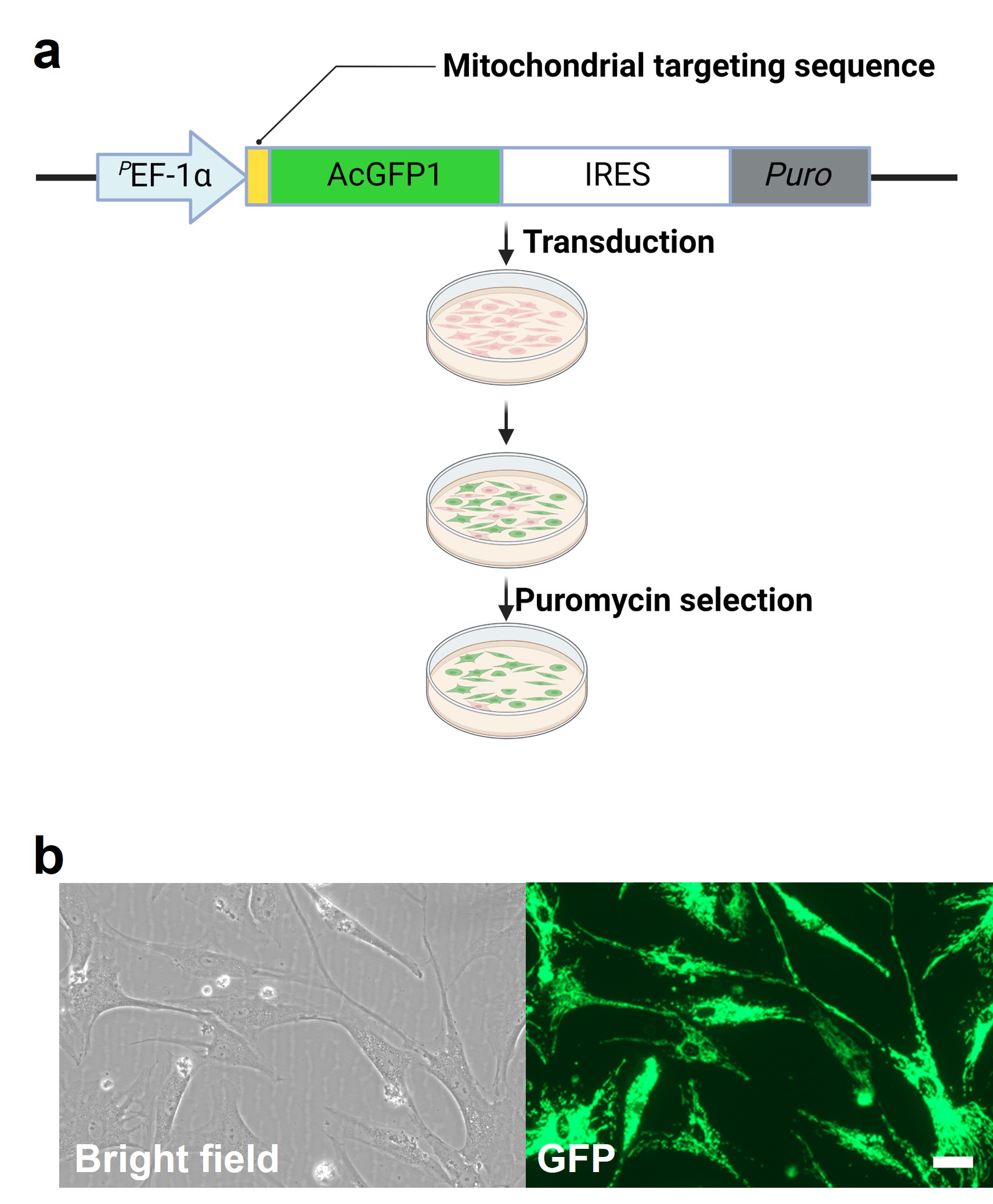


**Figure S18. Lentiviral construct for introduction of the AcGFP1 protein for specific expression in mitochondria.** a) Schematic summarizing the construction of the lentiviral vector for generation of AcGFP1-mitochondria labeled MSCs by lentiviral transduction. b) EVOS bright field and fluorescence microscopy images of AcGFP1-mitochondria (green) in MSCs. Scale bar = 25 µm.


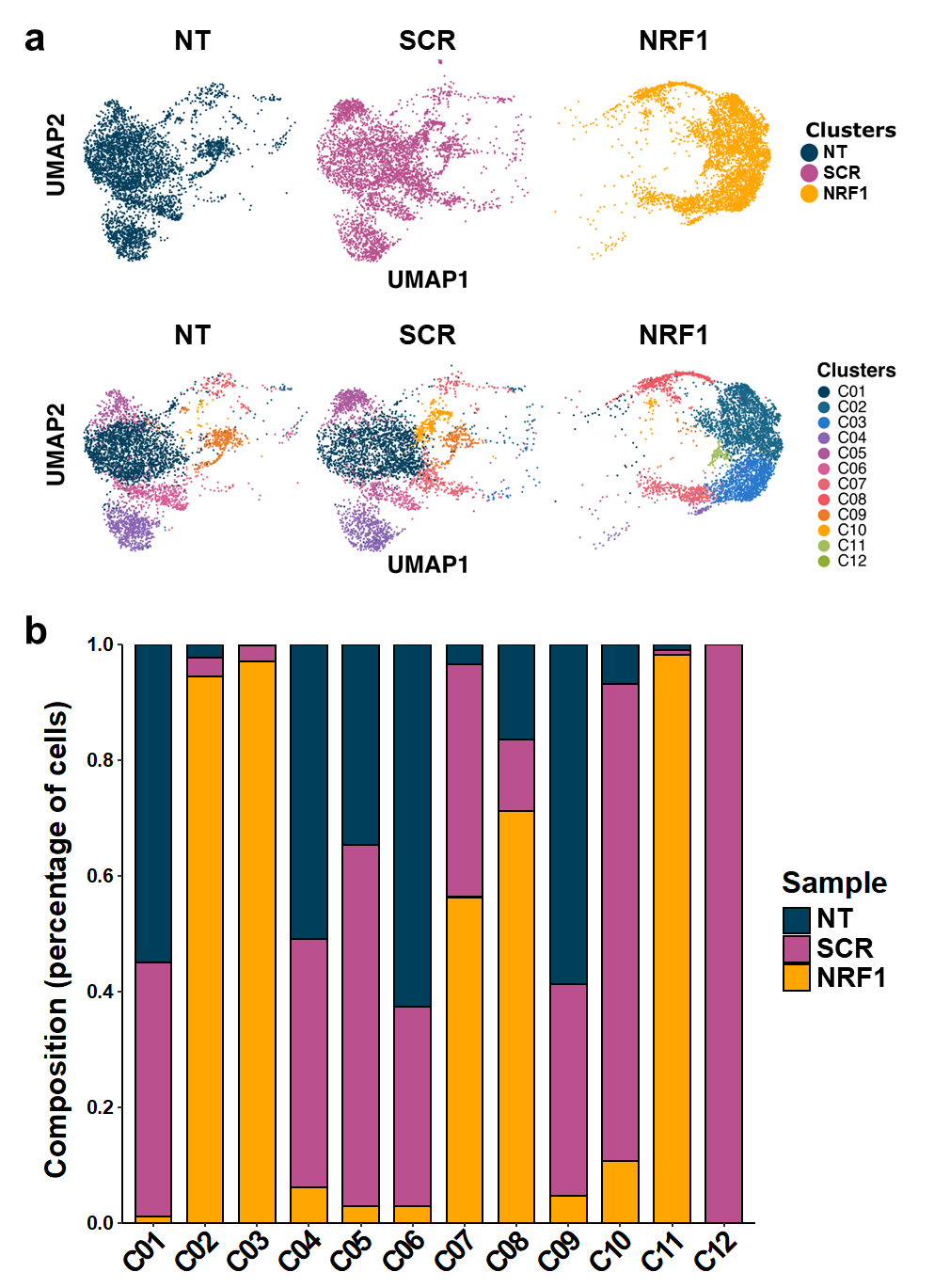


**Figure S19. Single MSC clustering for scRNA sequencing analysis. MSCs were transfected with either scrambled (SCR) or NRF1 mRNA.** Controls consisted of non-transfected MSCs (NT). a) UMAP plots of distinct subpopulations of cells consisting of control and SCR- or NRF1-mRNA transfected MSCs, resulting in the identification of 12 clusters by RNA expression pattern. b) Composition of clusters based on MSC treatment group.
